# PCR and Omics Based Techniques to Study the Diversity, Ecology and Biology of Anaerobic Fungi: Insights, Challenges and Opportunities

**DOI:** 10.3389/fmicb.2017.01657

**Published:** 2017-09-25

**Authors:** Joan E. Edwards, Robert J. Forster, Tony M. Callaghan, Veronika Dollhofer, Sumit S. Dagar, Yanfen Cheng, Jongsoo Chang, Sandra Kittelmann, Katerina Fliegerova, Anil K. Puniya, John K. Henske, Sean P. Gilmore, Michelle A. O'Malley, Gareth W. Griffith, Hauke Smidt

**Affiliations:** ^1^Laboratory of Microbiology, Wageningen University & Research Wageningen, Netherlands; ^2^Lethbridge Research and Development Centre, Agriculture and Agri-Food Canada Lethbridge, AB, Canada; ^3^Department for Quality Assurance and Analytics, Bavarian State Research Center for Agriculture Freising, Germany; ^4^Bioenergy Group, Agharkar Research Institute Pune, India; ^5^Laboratory of Gastrointestinal Microbiology, Nanjing Agricultural University Nanjing, China; ^6^Department of Agricultural Science, Korea National Open University Seoul, South Korea; ^7^Grasslands Research Centre, AgResearch Ltd. Palmerston North, New Zealand; ^8^Institute of Animal Physiology and Genetics, Czech Academy of Sciences Prague, Czechia; ^9^College of Dairy Science and Technology, Guru Angad Dev Veterinary and Animal Sciences University Ludhiana, India; ^10^Dairy Microbiology Division, ICAR-National Dairy Research Institute Karnal, India; ^11^Department of Chemical Engineering, University of California, Santa Barbara Santa Barbara, CA, United States; ^12^Institute of Biological Environmental and Rural Sciences, Aberystwyth University Aberystwyth, United Kingdom

**Keywords:** anaerobic fungi, Neocallimastigomycota, rumen, phylogeny, genomics, (meta) transcriptomics, proteomics, metabolomics

## Abstract

Anaerobic fungi (phylum Neocallimastigomycota) are common inhabitants of the digestive tract of mammalian herbivores, and in the rumen, can account for up to 20% of the microbial biomass. Anaerobic fungi play a primary role in the degradation of lignocellulosic plant material. They also have a syntrophic interaction with methanogenic archaea, which increases their fiber degradation activity. To date, nine anaerobic fungal genera have been described, with further novel taxonomic groupings known to exist based on culture-independent molecular surveys. However, the true extent of their diversity may be even more extensively underestimated as anaerobic fungi continue being discovered in yet unexplored gut and non-gut environments. Additionally many studies are now known to have used primers that provide incomplete coverage of the Neocallimastigomycota. For ecological studies the internal transcribed spacer 1 region (ITS1) has been the taxonomic marker of choice, but due to various limitations the large subunit rRNA (LSU) is now being increasingly used. How the continued expansion of our knowledge regarding anaerobic fungal diversity will impact on our understanding of their biology and ecological role remains unclear; particularly as it is becoming apparent that anaerobic fungi display niche differentiation. As a consequence, there is a need to move beyond the broad generalization of anaerobic fungi as fiber-degraders, and explore the fundamental differences that underpin their ability to exist in distinct ecological niches. Application of genomics, transcriptomics, proteomics and metabolomics to their study in pure/mixed cultures and environmental samples will be invaluable in this process. To date the genomes and transcriptomes of several characterized anaerobic fungal isolates have been successfully generated. In contrast, the application of proteomics and metabolomics to anaerobic fungal analysis is still in its infancy. A central problem for all analyses, however, is the limited functional annotation of anaerobic fungal sequence data. There is therefore an urgent need to expand information held within publicly available reference databases. Once this challenge is overcome, along with improved sample collection and extraction, the application of these techniques will be key in furthering our understanding of the ecological role and impact of anaerobic fungi in the wide range of environments they inhabit.

## Introduction

Anaerobic fungi (phylum Neocallimastigomycota) are the most effective fiber degrading microorganisms in the gut of mammalian herbivores. This is due to their combined mechanical and enzymatic activity, and the associated ability to penetrate plant structural barriers (Orpin, 1975; Ho et al., 1988; Lee et al., 1999). These attributes are underpinned during the vegetative and motile stages of their life cycle, with a putative aerotolerant resistant stage more associated with survival outside the mammalian gut (Davies et al., 1993; McGranaghan et al., 1999). During the first 40 years following their recognition as fungi by Orpin in 1975, only six genera of anaerobic fungi were named within phylum Neocallimastigomycota (*Anaeromyces, Caecomyces, Cyllamyces, Neocallimastix, Orpinomyces*, and *Piromyces*). Within the last 2 years three new genera of anaerobic fungi have been described: *Buwchfawromyces* with its type species *Buwchfawromyces eastonii* (Callaghan et al., 2015; formerly known as SK2 clade, Koetschan et al., 2014), *Oontomyces* with its type species *Oontomyces anksri* (Dagar et al., 2015), and *Pecoramyces* (formerly known as *Orpinomyces* sp. C1A; Youssef et al., 2013) with its type species *Pecoramyces ruminantium* (Hanafy et al., 2017).

Anaerobic fungi have been largely described in the past on the basis of their morphological characteristics. However, due to factors such as convergent evolution and highly variable *in vitro* growth characteristics, morphological features alone are not sufficient to distinguish between certain genera and species. Within the nine characterized genera more than 20 species have been described, however, genetic analysis indicated that despite different generic names, some species are identical. On the other hand, description of new species supported by morphological and genetic analysis has recently enlarged the group of cultivated anaerobic fungi (Ariyawansa et al., 2015; Li G. J., et al., 2016).

Anaerobic fungi have been most extensively studied in ruminants, but in recent years an increasing amount of anaerobic fungal research has focused on their biotechnological exploitation (Prochazka et al., 2012; Gruninger et al., 2014; Haitjema et al., 2014; Gilmore et al., 2015; Peng et al., 2016; Solomon et al., 2016a; Dollhofer et al., 2017). This has been mainly in terms of their potent fiber degrading enzymes, due to challenges with cultivating anaerobic fungi in large scale continuous systems limiting their direct application (Gruninger et al., 2014; Solomon et al., 2016a). For the anaerobic fungal research community, this biotechnological interest has led to substantial investment that has enabled valuable advances in anaerobic fungal knowledge and resources, particularly from genomic and transcriptomic data in recent years.

In this review, we focus on the different types of molecular methods, including ‘omics approaches, that have been used to date in the study of anaerobic fungi, and we highlight the challenges that currently exist—many of which are fundamentally different from those encountered with the more routinely studied rumen bacteria and archaea.

## Barcode markers for rapid phylotyping of anaerobic fungi

**Figure 1:**
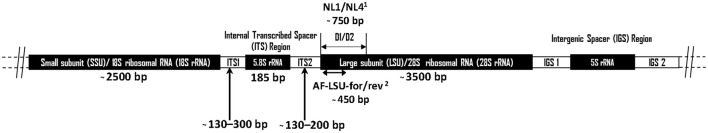
Schematic diagram showing the arrangement of the anaerobic fungal *rrn* operon. Size information on the ITS1 region was from Liggenstoffer et al. (2010) and ITS2 was based on 49 Genbank sequences. Sizes of other regions are based on a Genbank reference sequence (AJ864475). Primer references: ^1^White et al. (1990) and ^2^Dollhofer et al. (2016).

The “Assembling the Fungal Tree of Life” (AFToL) project used a multi-gene approach to decipher, to high resolution, the low level evolutionary phylogenetic relationships between the fungal Kingdom (James et al., 2006). The six genes used were those encoding 18S ribosomal RNA (rRNA), 28S rRNA, 5.8S rRNA, Elongation Factor 1-alpha (EF1α), and two RNA polymerase II subunits (RPB1 and RPB2). However, it is important to recognise the difference between barcoding loci and other loci suitable for phylogenetic inference. Potential barcoding loci, particularly those useful for next generation sequencing (NGS) based diversity studies, are those found on the multicopy *rrn* (rRNA) operon (Figure 1). These loci have a high copy number (ca. 200) per genome meaning only small amounts of tissue or environmental sample are needed for efficient PCR amplification. Additionally, these loci are not protein coding, therefore having a relatively high mutation rate enabling good phylogenetic resolution (Hibbett et al., 2007). Consequently, for barcoding and environmental sequencing studies loci belonging to the *rrn* operon are most suitable, thus such research into anaerobic fungi over the last 25 years has focussed on numerous different regions within this operon (Figure 1, Table 1).

**Table 1:** Details of different genes/regions and primers used for studying diversity and taxonomy of anaerobic rumen fungi.

**Target gene**	**Sample type**	**Primers (5′-3′)^a^**	**Application**	**References**
18S rRNA (SSU)	Pure culture	309e- TCAGGCTCCCTCTCCGG 519- GWATTACCGCGGCKGCTG 686e- AGAATTTCACCTCTG 926e- CCGTCAATTC(AC)TTT(AG)AGTTT 18J.CPM- CAGACACTACGGGAATCT 1400- ACGGGCGGTGTGT(GA)C 915-GCCCCCG(TC)CAATTCCT 920- ATTCCTTT(GA)AGTTT 956- GGCGTTGTGTC(CG)AATTAA 1100- AGGGTTGCGCTCGTT 1100a- TGGGTCTCGCTCGTTG 1511e- C(CT)GCAGGTTCACCTAC	Sequencing	Dore and Stahl, 1991
	Pure culture	SL2l (R)- CCGAATTCGTAGTCATATGCTTGTCT SL27 (F)- CCAAGCTTAAACCTTGTTACGACTT	Cloning and sequencing	Bowman et al., 1992
	Pure culture	NS1 (F)- GTAGTCATATGCTTGTCTC NS2 (R)- GGCTGCTGGCACCAGACTTGC	PCR-RFLP	Fliegerova et al., 2006
	Pure culture, rumen fluid and biogas plant sludge	AF-SSU forward- CTAGGGATCGGACGACGTTT AF-SSU reverse- GGACCTYCCGATCAAGGATG AF-SSU probe- 6FAM-ATTC GCGTAACTATTTAGCAGGTTAAGGT-BHQ1	qRT-PCR	Dollhofer et al., 2016
Internal transcribed spacer 1 (ITS1)	Pure culture	(F)- TGTACACACCGCCCGTC (R)- CTGCGTTCTTCATCGAT	Sequencing	Li and Heath, 1992
	Pure culture		Cloning and sequencing	Brookman et al., 2000
	Pure culture		PCR-RFLP	Hausner et al., 2000
	Pure culture	ITS 1 (F)- TCCGTAGGTGAACCTGCGG ITS 2 (R)- GCTGCGTTCTTCATCGATGC	PCR-RFLP	Fliegerova et al., 2002
	Pure culture	MN100 (F)- TCCTACCCTTTGTGAATTTG MNGM2 (R)- CTGCGTTCTTCATCGTTGCG	ITS1 fingerprinting	Tuckwell et al., 2005
	Pure culture	Neo 18S (F)- 6FAM-AATCCTTCGGATTGGCT Neo 5.8S (R)- CGAGAACCAAGAGATCCA	ARISA	Edwards et al., 2008
	Pure culture and rumen fluid	MN100 (F)- 6FAM-TCCTACCCTTTGTGAATTTG MNGM2 (R)-CTGCGTTCTTCATCGTTGCG	ARISA	Denman et al., 2008
	Rumen digesta	Neo 18S (F)- 6FAM-AATCCTTCGGATTGGCT Neo 5.8S (R)- CGAGAACCAAGAGATCCA	ARISA	Cheng et al., 2009
	Fecal samples	(F)- GCCTCCCTCGCGCCATCAG-(barcode)- TCCTACCCTTTGTGAATTTG (R)- GCCTTGCCAGCCCGCTCAG- CTGCGTTCTTCATCGTTGCG	Pyrosequencing	Liggenstoffer et al., 2010
	Pig and cow manure	ITS1F (F)- CTTGGTCATTTAGAGGAAGTAA Neo QPCR (R)- GTGCAATATGCGTTCGAAGATT	Cloning and sequencing	Fliegerova et al., 2010
	Fecal samples	MN100 (F)- TCCTACCCTTTGTGAATTTG MNGM2 (R)	PCR-DGGE	Nicholson et al., 2010
	Rumen fluid	CTGCGTTCTTCATCGTTGCGCGCCCGCCGCG CGCGGCGGGCGGGGCGGGGGCACGGGGGG	PCR-DGGE	Khejornsart and Wanapat, 2010
	Rumen fluid		PCR-DGGE	Khejornsart et al., 2011
	Rumen fluid and digesta		PCR-DGGE	Kittelmann et al., 2012
	Pure culture and rumen fluid	qRT-PCR-fungi (F)- GAGGAAGTAAAAGTCGTAACAAGGTTTC qRT-PCR-fungi (R)- CAAATTCACAAAGGGTAGGATGATT	qRT-PCR	Lwin et al., 2011
	Rumen fluid and digesta	MN100 (F)- TCCTACCCTTTGTGAATTTG MNGM2 (R)- CTGCGTTCTTCATCGTTGCG Adaptors A (CCATCTCATCCCTGCGTGTCTCCGACTCAG) or B (CCTATCCCCTGTGTGCCTTGGCAGTCTCAG)	Pyrosequencing	Kittelmann et al., 2012
	Rumen fluid and digesta	ITS1F (F)- TCCGTAGGTGAACCTGCGG ITS400Rw (R)- ATTGTCAAAAGTTGTTTTTAWATTAT	Cloning and sequencing	Kittelmann et al., 2012
5.8S rRNA	Pure culture and rumen digesta	Neo QPCR (F)-TTGACAATGGATCTCTTGGTTCTC Neo QPCR (R)- GTGCAATATGCGTTCGAAGATT Taqman probe: Neo 6FAM-CAAAATGCGATAAGTARTGTGAATT GCAGAATACG-TAMRA	qRT-PCR	Edwards et al., 2008
SSU and ITS1	Pure culture, rumen fluid and digesta	(F)- GAGGAAGTAAAAGTCGTAACAAGGTTTC (R)- CAAATTCACAAAGGGTAGGATGATT	qRT-PCR	Denman and Mcsweeney, 2006
	Rumen fluid and digesta		QC-PCR	Sekhavati et al., 2009
	Rumen fluid and digesta		qRT-PCR	Khejornsart et al., 2011
	Rumen fluid		qRT-PCR	Kittelmann et al., 2012
Internal transcribed spacer (ITS)	Pure culture	ITS 1 (F)- TCCGTAGGTGAACCTGCGG ITS 4 (R)- TCCTCCGCTTATTGATATGC	PCR-RFLP	Fliegerova et al., 2002, 2006
			Cloning and sequencing	Fliegerova et al., 2004
		JB206 (F)- GGAAGTAAAAGTCGTAACAAGG JB205 (R)- TCCTCCGCTTATTAATATGC	Cloning and sequencing	Tuckwell et al., 2005
		GM1 (F)- TGTACACACCGCCCGTC JB205 (R)- TCCTCCGCTTATTAATATGC	Cloning and sequencing	Nicholson et al., 2010
		ITS1F (F)- CTTGGTCATTTAGAGGAAGTAA EminITS4 (R)- GTTCAGCGGGTACTCTTATCTG	PCR-RFLP	Griffith et al., 2009
		JB206 (F)- GGAAGTAAAAGTCGTAACAAGG JB205 (R)- TCCTCCGCTTATTAATATGC	Cloning and sequencing	Solomon et al., 2016a
28S rRNA (LSU)	Pure culture	(F)- GCCTTAGTAACGGCGAGTG (R)- GGAACCTTTCCCCACTTC	PCR-RFLP	Hausner et al., 2000
		NL1 (F)- GCATATCAATAAGCGGAGGAAAAG NL4 (R)- GGTCCGTGTTTCAAGACGG	PCR-RFLP	Fliegerova et al., 2006
			PCR-RFLP	Dagar et al., 2011, 2014
	Pure culture, rumen fluid and biogas plant sludge	AF-LSU (F)- GCTCAAAYTTGAAATCTTMAAG AF-LSU (R)- CTTGTTAAMYRAAAAGTGCATT	Cloning and sequencing	Dollhofer et al., 2016
ITS and LSU	Pure culture	ITS5 (F)- GGAAGTAAAAGTCGTAACAAGG NL4 (R)- GGTCCGTGTTTCAAGACGG	Cloning and sequencing	Wang et al., 2017
Intergenic spacer region (IGS)	Pure culture	(F)- GAGACAAGCATATGACTAC (R)- ACGCCTCTAAGTCAGAAT	PCR-RFLP	Hausner et al., 2000
GH5 cellulolytic endoglucanase	Pure culture, rumen fluid and biogas plant sludge	AF-Endo (F)- CGTATTCCAACYACTTGGWSYGG AF-Endo (R)- CCRKTRTTTAAGGCAAARTTRTAYGGA	qRT-PCR	Dollhofer et al., 2016

a *The use of the primer in a forward (F) or reverse (R) orientation is indicated, with exception of the primers from Dore and Stahl (1991) that were used to sequence RNA*.

Contrasting with this, taxonomic loci tend to be single copy protein coding genes, including e.g., RPB1 and RPB2 (James et al., 2006) and EF-1α (Eckart et al., 2010). These genes code for critically important functional proteins. Hence, mutations across these genes are likely to cause a loss of fitness or death of the organism, and as a result these genes are highly conserved. Interestingly, it has been found that anaerobic fungi have two paralogous copies of EF-1α (Eckart et al., 2010), which is perhaps not that surprising considering the large amount of repetition that has been reported to occur in anaerobic fungal genomes (Haitjema et al., 2017). This phenomenon has also been reported to occur in other basal fungal taxa (James et al., 2006), and limits the value of this gene as a marker. Single copy protein encoding genes enable reliable higher level phylogenetic classification, but are not so useful in differentiating closely related fungi to the species level.

The small-subunit (SSU) rRNA gene is widely used as a barcode marker for bacteria, archaea and protists, and has also been looked at as a barcoding and quantification loci for the anaerobic fungi (Dore and Stahl, 1991; Brookman et al., 2000; Dollhofer et al., 2016). The much shorter 5.8S rRNA (185 bp) has also been used in qPCR based quantification methods for anaerobic fungi (Edwards et al., 2008). However, neither 18S nor 5.8S rRNA loci are variable enough to enable phylogenetic differentiation between all anaerobic fungal genera (Eckart et al., 2010; Dollhofer et al., 2016). Therefore, the internal transcribed spacer 1 (ITS1) region has instead been used most extensively for differentiating genera and species of anaerobic fungi, and has been widely applied to the study of anaerobic fungi in a range of mammalian herbivores (Table 1). In this section, the current state of the art regarding barcoding loci for anaerobic fungi is reviewed, and the reasons behind the recent move within the research community toward the use of the large subunit (LSU) 28S rRNA as a barcoding locus are highlighted.

### Internal transcribed spacer region

The ITS region is the barcode of choice for the fungal kingdom (Schoch et al., 2012), and has also been widely used for the identification of anaerobic fungi in culture and environmental surveys (Table 1). To date, molecular identification of anaerobic fungi in culture has mainly been done using Sanger sequencing, which can cope well with the AT richness of the ITS1 region. ITS1 has also proven highly useful in molecular surveys that evaluated the diversity and community structure of anaerobic fungi in different environments or hosts based on clone libraries (Fliegerova et al., 2010; Nicholson et al., 2010; Kittelmann et al., 2012).

Over the past decade, next-generation sequencing of the ITS1 region has allowed large-scale analysis of anaerobic fungal diversity and community structure in various host animals (Liggenstoffer et al., 2010; Kittelmann et al., 2013). However, the large number of sequences obtained does not allow for tree-based evaluation of individual sequences, with OTU based methods used instead (Liggenstoffer et al., 2010; Kittelmann et al., 2013). Limited length of sequence reads also restricts the ability to generate a reliable phylogenetic analysis, particularly due to the large size polymorphism that exists for the anaerobic fungal ITS1 region. As a consequence, sequences representative of the OTUs are instead assigned taxonomic classifications through sequence similarity (BLAST) searches against public databases such as NCBI's GenBank (Benson et al., 2013) or more specific ITS databases, such as UNITE (Kõljalg et al., 2005; Abarenkov et al., 2010) or ITSoneDB (Santamaria et al., 2012). The quality of these databases, however, strongly depends on the quantity of relevant content and scientific rigor of contributors, and the more comprehensive the database, the more challenging is the task of regular manual curation. Thus, it is not surprising that in the past large numbers of anaerobic fungal sequences in GenBank have been found to be misnamed at the genus level (Fliegerova et al., 2010; Kittelmann et al., 2012). This significantly jeopardizes the interpretation of sequence data. These shortcomings highlight the need for a more curated approach for taxonomic analysis of anaerobic fungal sequence data within the research community. This would ideally be guided by a stable anaerobic fungal phylogeny where reference genomes are fully sequenced, with uncharacterised classifications (i.e., unclassified Neocallimastigales) at higher taxonomic ranks avoided (Kittelmann et al., 2012).

Molecular surveys based on the ITS1 marker have suggested the existence of several novel anaerobic fungal clades, but their relatedness to known genera remained inconclusive due to the lack of a stable ITS1 phylogeny (Fliegerova et al., 2010; Liggenstoffer et al., 2010; Nicholson et al., 2010; Herrera et al., 2011; Kittelmann et al., 2012). The instability of the ITS1 phylogeny is primarily caused by difficulties with aligning this polymorphic and homoplasious region. Whilst issues with ITS1 heterogeneity cannot be easily overcome, the use of secondary structure information can be used to improve the analysis of ITS1 sequence data by enabling structure-informed sequence alignments.

Using secondary structure information, Tuckwell et al. (2005) defined four variable regions within the ITS1 of the anaerobic fungi, and generated diagnostic fingerprints for the different genera. More recently, Koetschan et al. (2014) suggested a common secondary core structure for the ITS1 of the anaerobic fungi, and developed an automated folding and alignment approach. For the ITS2, this method had previously enabled its use even for elucidating high level phylogenetic relationships (Coleman, 2003; Buchheim et al., 2011a,b), resulting in significantly more robust and more accurate tree reconstructions (Keller et al., 2010). Similarly, for the ITS1, both primary sequence and secondary structure information now guide automated sequence alignment using the 4SALE software (Seibel et al., 2006, 2008) as well as phylogenetic analysis with ProfDistS (Wolf et al., 2008), allowing for the calculation of a more stable anaerobic fungal ITS1 phylogeny (Koetschan et al., 2014).

**Figure 2:**
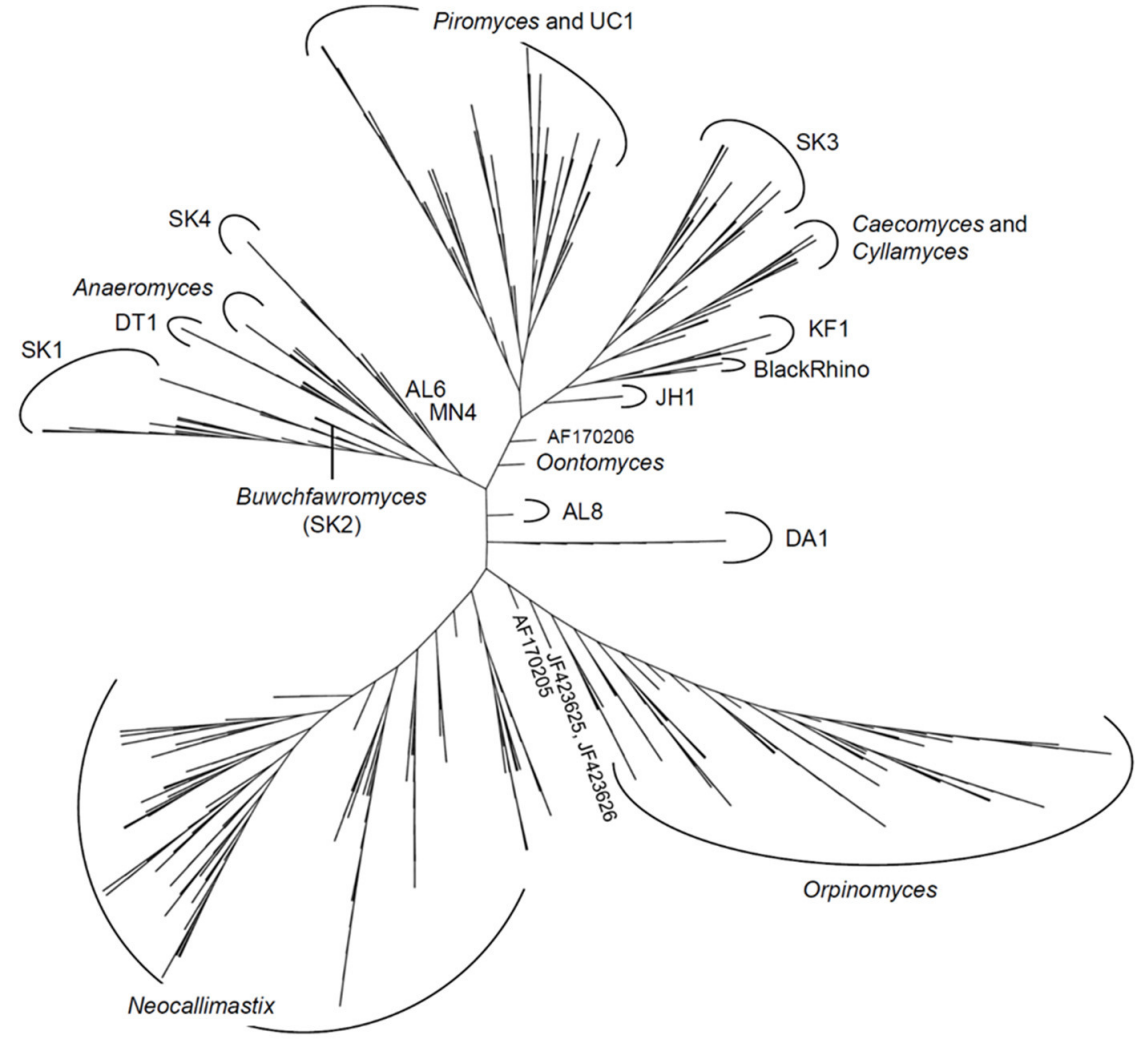
Phylogenetic tree of the anaerobic fungi (Neocallimastigomycota). The Profile Neighbor Joining tree was constructed according to (Koetschan et al., 2014) using a total of 576 unique Neocallimastigomycota ITS1 sequences and secondary structures [575 sequences used in Koetschan et al. (2014), and the reference sequence of *Oontomyces anksri* (Dagar et al., 2015)]. Sequences specified by GenBank accession numbers have not yet been assigned to any genus or clade. In addition to the eight named genera indicated (*Anaeromyces, Buwchfawromyces, Caecomyces, Cyllamyces, Neocallimastix, Oontomyces, Orpinomyces*, and *Piromyces*), the tree consists of at least 12 further monophyletic clades, which at present have no cultured representatives. This tree is reprinted with permission from Kittelmann et al. (2017), and copyright information is provided in the Acknowledgements.

The latest version of the ITS1 phylogeny according to Koetschan et al. (2014) classifies the anaerobic fungi into eight genera and 12 as yet uncultured genus- or species-level clades (Figure 2, *P. ruminantium* is not shown). The corresponding sequence database and taxonomy files (including the ITS1 sequence of *P. ruminantium*; available from the Anaerobic Fungi Network webpage, https://www.anaerobicfungi.org) are compatible with sequence analysis pipelines such as mothur (Schloss et al., 2009) and QIIME (Caporaso et al., 2010) and allow highly resolved taxonomic assignment of (next-generation) sequence data. Due to new data emerging, and clades being formally named according to newly isolated representatives (Callaghan et al., 2015; Hanafy et al., 2017), the database is being curated on a regular basis. It is likely that even further novel clades may exist, particularly as it is now recognized that many of the anaerobic fungal ITS1 primer sets used to date are not comprehensive (Callaghan et al., 2015). Based on available sequence information, the complete anaerobic fungal ITS1 region can be successfully amplified for all anaerobic fungi using either of the following primer pairs that both target the end of the 18S rRNA gene and the start of the 5.8S rRNA gene: Neo18S/Neo5.8 (Edwards et al., 2008) or ITS1F/ITS400Rw (Kittelmann et al., 2012) (see Table 1 for primer sequence details).

Despite the usefulness of ITS1 as a barcode marker to date, it is becoming increasingly apparent that its application as a phylogenetic marker has fundamental limitations. Within a single culture multiple cloned ITS1 sequences can vary as much as 13% between ITS1 repeats (Callaghan et al., 2015), and the ITS1 region itself can be variable in size (Edwards et al., 2008). Consequently, it can be difficult to differentiate whether a novel environmental ITS1 sequence type does indeed represent a new species/genus. Due to this, there has been a move in recent years to explore the potential of using LSU rRNA as a barcoding locus. The ITS1, however, will remain an important barcode marker for identifying anaerobic fungi, especially in environmental surveys that aim to characterize the entire mycobiome, including the Neocallimastigomycota, in a given sample (Belila et al., 2017). Therefore, the availability of a curated ITS1 database will be of particular importance for taxonomic identification of anaerobic fungi in novel host- and non-host associated habitats that are shared with other fungal taxa.

### Large sub-unit rRNA

The LSU rRNA gene is the longest of the *rrn* loci and codes for the 28S rRNA ribosomal sub-unit, which is approximately 3,500 bp long in the anaerobic fungi. The upstream 5′ region next to the ITS2 (Figure 1), known as the D1/D2 region, is commonly used in fungal barcoding studies as it provides significant variability that can discriminate phylotypes (Fell et al., 2000; Dagar et al., 2011; Schoch et al., 2012; Detheridge et al., 2016). Additionally, flanking regions are significantly conserved so that universal fungal (White et al., 1990; Detheridge et al., 2016) or group specific (Dollhofer et al., 2016) primers can be designed. This region also shows limited size variation among different genera and unlike ITS1 has limited intra-genomic sequence variation (apparent as SNPs in Sanger sequencing chromatograms; Callaghan, 2014). This makes alignment of these sequences straight forward compared to those from the ITS1 region.

Hausner et al. (2000) was the first to publish a 28S rRNA gene-targeted PCR-RFLP based method for anaerobic fungi, however, the large 1.65 kb PCR amplicon used had limited value for restriction characterization due to the presence of multiple restriction sites. Subsequently Fliegerova et al. (2006) successfully used a smaller amplicon spanning only the D1/D2 region of the anaerobic fungal LSU, using the universal fungal primers NL1/NL4 (White et al., 1990). Later, the same region and method were shown to be able to differentiate between two closely related anaerobic fungal species (Dagar et al., 2011), hinting at its potential value as a barcoding locus for the anaerobic fungi. Callaghan et al. (2015) used phylogenetic comparison of both the LSU and ITS1 region to classify the anaerobic fungal genus *Buwchfawromyces*. This study also contained a phylogenetic tree based upon the D1/D2 LSU region, which showed that all included genera and species could be resolved.

Dollhofer et al. (2016) published an amalgamated LSU tree (containing Genbank sequences and environmental clones) that was constructed using a 447 bp region of LSU D1/D2 amplified using anaerobic fungal specific primers (Table 1). This truncated amplicon (compared to NL1/NL4) still was sufficient to resolve sequences to genus and species level, and is therefore a good candidate region for future anaerobic fungal NGS studies (Dollhofer et al., 2016). As with the sequencing of any barcoding loci, the use of a high fidelity NGS platform is crucial. However, due to the size of the LSU D1/D2 amplicon (~450 bp) it is clear that a NGS platform also able to provide a reasonable overlap of the paired end reads of this amplicon is needed (i.e., 2 × 300 b), as sequence quality deteriorates toward the end of a read.

A recent paper comparing ITS1 and LSU based phylogenies concluded that sequences from LSU aligned easier and were better for distinguishing the different genera of anaerobic fungi than ITS1, although both LSU and ITS1 based phylogenies showed a high degree of similarity (Wang et al., 2017). Based on the limited number of available LSU sequences from *Caecomyces* and *Cyllamyces*, however, it was not clear if LSU could resolve these bulbous genera (Wang et al., 2017). Whether these two bulbous genera do indeed represent one single (Gruninger et al., 2014; Callaghan et al., 2015) or two (Ozkose et al., 2001) phylogenetically distinct clades though has recently been queried (Hanafy et al., 2017).

**Figure 3:**
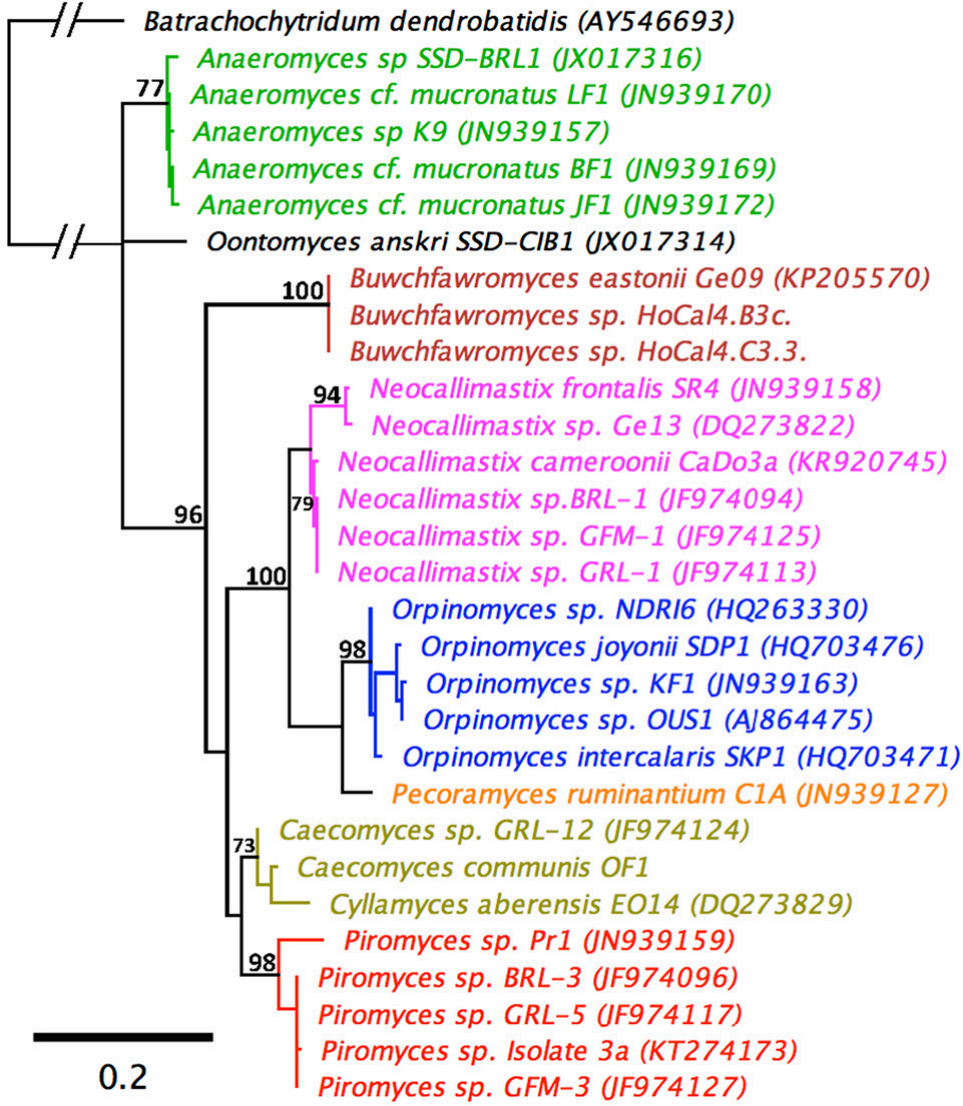
Maximum Likelihood phylogenetic tree based on a 744 bp alignment of 29 anaerobic fungal 28S rRNA gene sequences. The sequences are representative of all described genera. An aerobic chytrid *Batrachochytrium dendrobatidis* was used to root the tree. Topologies are sorted to over 70% (bootstrap = 1,000 replicates) and bootstrap values over 70% are shown. Scale bar shows substitutions per site. The different genera are color coded: *Anaeromyces* (green), *Buwchfawromyces* (brown), *Caecomyces*, and *Cyllamyces* (olive), *Neocallimastix* (pink), *Oontomyces* (black), *Orpinomyces* (blue), *Pecoramyces* (orange), and *Piromyces* (red).

An LSU based taxonomy made using all available Genbank sequences from pure anaerobic fungal cultures is shown in Figure 3. The phylogenetic tree shows sequences from all known genera and species are resolved and suggests that the monoflagellated *Oontomyces, Anaeromyces, Buwchfawromyces, Caecomyces, Cyllamyces*, and *Piromyces* form the basal linages. While the polyflagellated *Orpinomyces* and *Neocallimastix* genera group together with the monoflagellated *Pecoramyces*, and form the distal groups (Figure 3).

Together with definite improvements in creating alignments and the limited size and sequence heterogeneity of the LSU within a single culture, the LSU is now likely to become the new barcoding locus of choice for anaerobic fungal specific NGS studies (Callaghan et al., 2015; Dollhofer et al., 2016; Wang et al., 2017). The next challenge for the research community will then be how to connect existing and newly generated ITS1 data with the LSU based data. Targeted sequencing of cultured type strains will be key to achieving this objective. Another issue, however, is matching currently uncultivated taxa. Targeted sequencing of larger segments of the ribosomal operon is one strategy that could be used, and metagenomic studies, especially those employing NGS technology that generates longer reads, may also prove valuable in the future to provide this information. The best way to truly resolve fungal phylogeny with certainty, however, is to utilize markers over the entire genome (Grigoriev et al., 2014). This is becoming increasingly feasible for anaerobic fungi in light of recent (meta-)genomic developments within the research community, as further detailed in the following section.

## Genomics

Whilst genetic markers have value in enabling molecular tools to be developed and establishing a taxonomic structure, it is the genomes that provide a key foundation in terms of understanding anaerobic fungal biology. The first thorough analysis of the genic, intergenic and rRNA encoding regions of a variety of genomic segments of an anaerobic fungus was performed by Nicholson et al. (2005) using directed plasmid libraries. The study provided observations on rules governing intron boundaries, the codon biases observed with different types of genes, and the sequence of an anaerobic fungal promoter. However, anaerobic fungal genomes have proven notoriously difficult to sequence due to their high AT-content, repeat-richness, complex physiology and unknown ploidy. Since this initial study, five anaerobic fungal genomes are now published and publically available, which has been the direct result of improvements in long-read sequencing technologies to overcome the aforementioned issues: *Piromyces* sp. E2, *P. ruminantium* C1A (formerly *Orpinomyce*s sp. C1A), *Neocallimastix californiae, Piromyces finnis*, and *Anaeromyces robustus* (Youssef et al., 2013; Haitjema et al., 2017). Despite this, however, the methods employed and/or developed in the process of generating these genomes have been found to be not always successful when applied to other anaerobic fungi within the research community. As a result, many recent efforts have failed either in terms of (i) generating high enough quality genomic DNA, (ii) assembling short read sequence data or (iii) annotating newly sequenced genomes. In this section, we review the practical considerations and current challenges faced when generating and analyzing anaerobic fungal genomes, as well as highlighting the valuable insights that have already been gained to date.

### Requirements for culturing and genomic DNA isolation

The major requirement for successful genome sequencing is high molecular weight (>10 kb) DNA in high quality (no RNA, protein or carbohydrate impurities) and quantity (>12 μg). With anaerobic fungi, several issues hinder researchers, when fulfilling these requirements. Anaerobic fungal cells are protected by a thick, recalcitrant cell wall containing chitin (Orpin, 1977), which is resistant to degradation by microbes and conventional cell lysis procedures. Mechanical treatments like freeze-drying, grinding in liquid N_2_, or bead beating are effective strategies to break open anaerobic fungal cells, and have been recommended to be performed prior to the application of chemical or kit based extraction methods (Solomon et al., 2016b). Mechanical treatments should be performed cautiously, however, as intense mechanical disruption can cause heat-induced DNA shearing resulting in low molecular weight DNA. Additional enzymatic lysis may also be included in the extraction work flow but to date only lyticase treatment has been reported to improve DNA yield and purity (Solomon et al., 2016b).

After overcoming the barrier of the anaerobic fungal cell wall, persistent RNA, protein and carbohydrate residues are a significant challenge when purifying the genomic DNA. Solomon et al. (2016b) compared several chemical and kit based extraction methods, including the cetyltrimethylammonium bromide (CTAB) protocol used by Youssef et al. (2013) prior to successful genome sequencing of *P. ruminantium* C1A. The commercially available PowerPlant® Pro DNA isolation kit alongside the CTAB method used by Youssef et al. (2013) were identified to deliver the best results for isolates from the *Piromyces, Neocallimastix*, and *Anaeromyces* genera. Within the research community, however, numerous researchers have faced challenges with isolating DNA of suitable quality and/or molecular weight, particularly from bulbous fungi, despite using one or more of these approaches.

As well as the DNA extraction method, the culture conditions used to generate the anaerobic fungal biomass can also influence the effectiveness of nucleic acid extractions and the interpretability of results. For high DNA yields, anaerobic fungi should be actively growing, thus to date incubation times from 3 to 4 days leading to cultures in mid-log to late-log-phase were used (Youssef et al., 2013; Solomon et al., 2016a). Strategies regarding culture volumes ranged from pooling several smaller parallel cultures (which seems to be the more efficient technique) up to 1-2 L cultures. For future experiments harvesting zoospores (Calkins et al., 2016) could become a potential starting point for nucleic acid extractions, potentially simplifying extraction procedures as this particular growth phase lacks a recalcitrant cell wall. This would also enable scientists to compare the genomes & epigenomes of different anaerobic fungi in a more standardized way, as all the cells would be more certain of being in a similar growth state. In order to do this most effectively, however, it is necessary to “synchronize” cultures as for example has been previously done with *Saccharomyces cerevisiae* (Hur et al., 2011).

For genome assembly, the presence of small contaminating DNA fragments in rumen fluid containing media has been considered a practical challenge. Some researchers have thus turned to rumen fluid free basal media (as described by Lowe et al., 1985) in combination with antimicrobial agents (penicillin, streptomycin, and chloramphenicol; Youssef et al., 2013) to eliminate background DNA and potential contamination by DNA originating from methanogens and bacteria. These small contaminating DNA fragments, however, can easily be removed through the use of DNA size selection that is typical of a long-read PacBio Single Molecular Real-Time (SMRT) sequencing library preparation. For example, Haitjema et al. (2017) employed BluePippin purification to select only high molecular weight (>10 kb) DNA fragments for genome sequencing of *P. finnis, N. californiae*, and *A. robustus*. This process removed contaminating DNA present in the rumen fluid that is typically present as small fragments, particularly after autoclaving media for sterility. To further improve DNA isolation and purity, media containing soluble sugars (e.g., cellobiose and glucose) rather than fibrous plant material have been employed (Youssef et al., 2013; Haitjema et al., 2017). Whilst these growth conditions have proven successful for all sequenced anaerobic fungi to date, it is yet to be seen if this cultivation approach can be universally applied.

### Sequencing, assembly, and annotation

For the genome analysis of *Piromyces* species E2, the first anaerobic fungal genome sequenced (made public in 2011), Sanger sequencing (read length 800–900 bp) in combination with Illumina Solexa (read length 2 × 75 bp) sequencing was employed followed by assembly with the use of Velvet (Haitjema et al., 2017). Due to the short reads generated with the sequencing techniques used, only a fragmented assembly with 39.7% of scaffolds representing gaps and high contig number was achieved. Similar results were observed with the genome sequencing of *P. ruminantium* C1A by Youssef et al. (2013) when only an Illumina 100 bp paired-end sequencing approach on a HiSeq 2000 approach was applied. The derived reads were not sufficient for whole genome assembly, as the resulting assembly (also done with Velvet) was highly fragmented with 82,325 contigs of which 32.4% were very short. To overcome these issues Single Molecule Real-Time (SMRT) sequencing with an average read length of 2,124 bp on a PacBio RS sequencing platform was performed (Youssef et al., 2013). The combination of both data sets lead to a non-fragmented final assembly allowing identification of large additional introns not detected when only using the Illumina data. The low GC (8.1%) content in the respective sequences and the frequent occurrence of microsatellites is likely to have led to the earlier lack of detection (Ross et al., 2013).

For the most recently sequenced genomes, of the species *N. californiae, Pir. Finnis*, and *A. robustus*, only PacBio SMRT sequencing was performed with high molecular weight DNA fragments (>10 kb), which yielded far improved genome assemblies, and the highest quality anaerobic fungal genomes reported to date (Haitjema et al., 2017). Assembly with Falcon (https://github.com/PacificBiosciences/FALCON), FinisherSC (Lam et al., 2015) and Quiver (https://github.com/PacificBiosciences/GenomicConsensus) generated even better assemblies compared to the hybrid Illumina-SMRT approach used by Youssef et al. (2013). This improvement is likely a result of improved isolation of high molecular weight DNA and sequencing of larger fragments. A comparison of all currently available gut fungal genome assemblies is presented in Table 2. Due to the long-read sequence technology, SMRT sequencing on PacBio is currently the gold standard platform for sequencing anaerobic fungal genomes, being capable of sequencing low GC content genomes and delivering non-fragmented final assemblies with low contig number and superior scaffold length. As such, a number of novel anaerobic fungal isolates are currently in the queue awaiting genome sequencing via PacBio at the DOE-JGI to increase the pool of high-quality genomic assemblies.

**Table 2:** Summary statistics for the anaerobic fungal genomes assembled to date (modified from http://genome.jgi.doe.gov/Pirfi3/Pirfi3.info.html).

**Genome assembly**	***Piromyces* sp. E2**	***Pecoramyces ruminantium* C1A^a^**	***Anaeromyces robustus***	***Neocallimastix californiae***	***Piromyces finnis***
Sequencing platform	Sanger & Illumina Solexa	PacBio SMRT & HiSeq 2000 sequencing platform, Illumina 100 bp paired-end	PacBio SMRT	PacBio SMRT	PacBio SMRT
Assembler	Velvet	Velvet & Whole Genome Shotgun Assembler	Falcon, FinisherSC, Quiver	Falcon, FinisherSC, Quiver	Falcon, FinisherSC, Quiver
Annotation	DOE-JGI Annotation Pipeline^*^ & Hidden Markov Modeling	BLASTP, BLASTX, NR database, HMMR Suite, PFAM database, IMG	DOE-JGI Annotation Pipeline^*^ & Hidden Markov Modeling	DOE-JGI Annotation Pipeline^*^ & Hidden Markov Modeling	DOE-JGI Annotation Pipeline^*^ & Hidden Markov Modeling
Genome assembly size (Mbp)	71.02	100.95	71.69	193.03	56.46
Sequencing read coverage depth	median ~6x, mean ~20x	300x	20x	20x	NA
# of contigs	17,217	32,574	1,035	1,819	232
Three largest Scaffolds (Mbp)	0.84, 0.64, 0.64	0.02, 0.02, 0.02	0.67, 0.50, 0.46	1.84, 1.45, 1.35	2.63, 1.96, 1.65

a *Formerly known as Orpinomyces sp. C1A*.

* *As described on the US Department of Energy Joint Genome Institute fungal portal MycoCosm (http://genome.jgi.doe.gov/programs/fungi/FungalGenomeAnnotationSOP.pdf)*.

**Figure 4:**
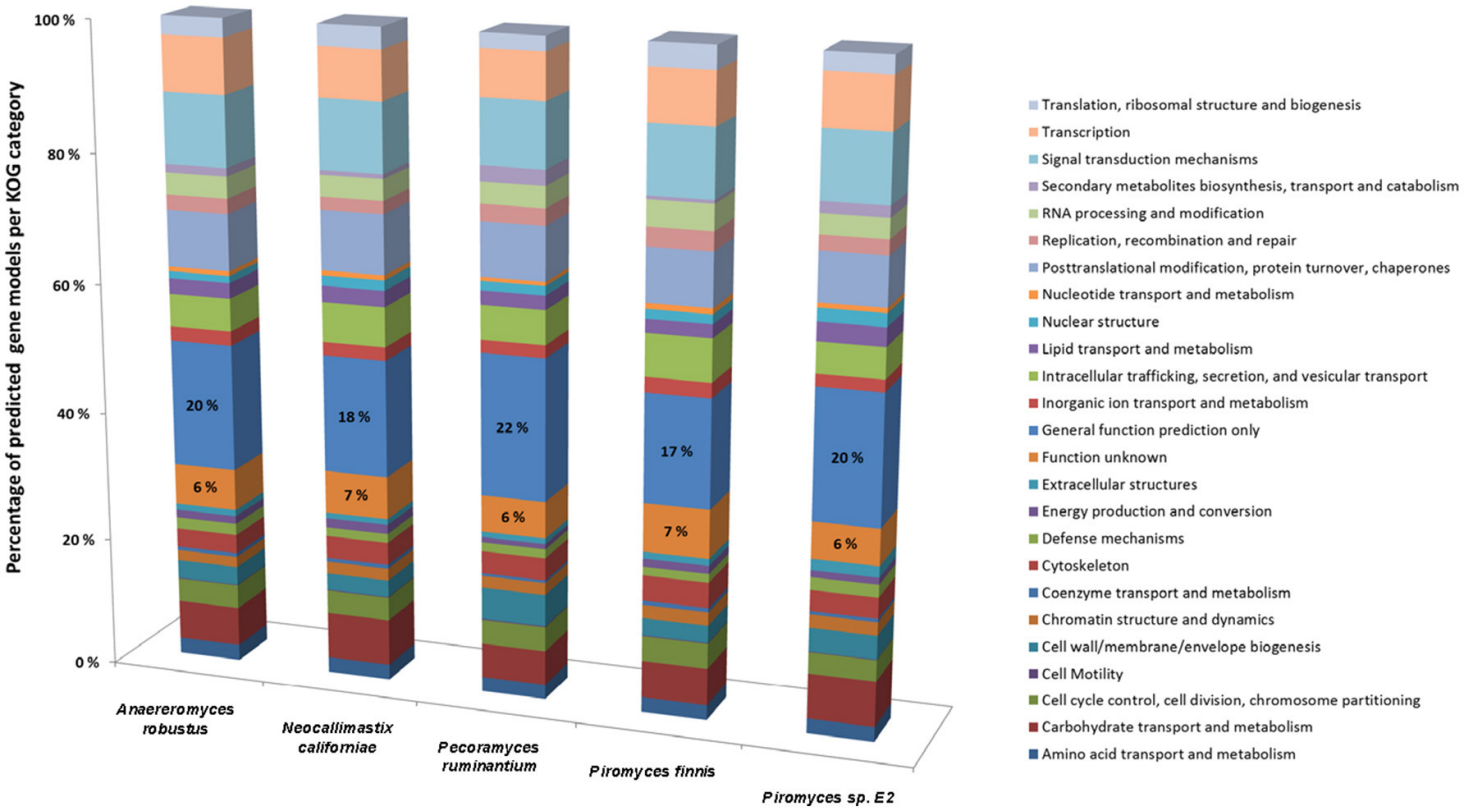
Comparison of gene models identified by KOG classification in the anaerobic fungal genomes sequenced to date. Percentages of the predicted gene models identified are shown due to the differences in genome assembly size between the organisms (see Table 2), with the percentage values for two categories (Function unknown & General function prediction only) also directly stated on the bars. Data sourced from Youssef et al. (2013) and Haitjema et al. (2017).

Whilst it is now possible to generate high quality anaerobic fungal genomes, it remains a challenge to assign correct functional annotations to novel anaerobic fungal genes. This becomes obvious, when the KOG data for all the available sequenced anaerobic fungal genomes is compared (Figure 4). On average 6% of the gene functions remained unknown and for 19% only a general function prediction was possible. Therefore, no function can be concisely described for 25% of the anaerobic fungal genes. Due to the lack of anaerobic fungal gene content in existing databases (e.g., KEGG and PFAM); accurate annotation of function is likely to remain a key challenge until this has been addressed. Interestingly, a recent study by Mondo et al. (2017) used epigenetic tools as a means to improve gene annotation across fungi. In particular, they showed that the early branching fungal lineage (including the anaerobic fungi) displayed unusual methylation islands (N6-methyldeoxyadenine) at transcriptional start sites of expressed genes (Mondo et al., 2017). As the ability to detect epigenetic modifiers (e.g., via PacBio) continues to improve, such tools will likely become invaluable to studying fungal genomes for which KOG, KEGG, and PFAM data are lacking.

Despite these functional annotation challenges, however, analysis of the genome of *P. ruminantium* C1A by conventional annotation strategies (see Table 2) discovered genomic traits potentially specific for the Neocallimastigomycota phylum and adapted to their anaerobic life-style (Youssef et al., 2013). Reconstruction of the *Pecoramyces* hydrogenosome allows the metabolism of energy production in anaerobic fungi to be clearly followed, revealing an incomplete tricarboxylic acid cycle and mixed-acid fermentation dependent energy formation. Characterization of the carbohydrate active enzymes (CAZymes) in *P. ruminantium* C1A also showed a huge and diverse range of lignocellulolytic genes, comprising 357 glycoside hydrolases (GHs), 24 polysaccharide lyases (PLs) and 92 carbohydrate esterases (CEs). From the genome of *Fibrobacter succinogenes*, a rumen bacterium specialized in lignocellulose utilization, 95 GHs, 5 PLs and 17 CEs were characterized, highlighting the extensive carbohydrate active enzymatic systems possessed by *P. ruminantium* C1A.

More recently, additional insights into anaerobic fungal CAZymes were generated from the analysis of four high quality anaerobic fungal genomes: *Piromyces* sp. E2, *N. californiae, P. finnis*, and *A. robustus* (Haitjema et al., 2017; genomes available via the Mycocosm website http://genome.jgi.doe.gov/programs/fungi/index.jsf). As expected, a wealth of CAZyme domains were identified across different regions of the genome, including hundreds of non-catalytic dockerin domain (NCDD) containing proteins native to the anaerobic fungi (Haitjema et al., 2017). Such domains are associated with fungal cellulosomes, which are multi-enzyme complexes produced to accelerate lignocellulose degradation (Gilmore et al., 2015). In particular, development of Hidden Markov Models (HMMs) based on integrated proteomic data obtained for these strains revealed a large, non-catalytic protein domain unique to the anaerobic fungi, later identified as scaffoldin domains for fungal cellulosomes (Haitjema et al., 2017). The high resolution of these anaerobic fungal genomes also enabled comparative genomic analyses, which for the first time quantified the frequency of horizontal gene transfer of CAZyme domains from anaerobic bacteria that are also resident in the rumen environment (Haitjema et al., 2017). Overall, these genomes have laid the foundation to interpret not only metabolic behavior of the anaerobic fungi, but also unique metabolites likely to be produced by these organisms—presumably to fine tune their interaction with other microbes within the rumen microbiome.

### Metagenomics

The rumen microbial community is a consortium of bacteria, archaea, anaerobic fungi, and protozoa. Bacteria and archaea represent the major proportion of microbes in terms of cell count, however, the eukaryotic anaerobic fungi and protozoa also represent a large proportion in terms of microbial biomass. To date, most rumen metagenomics studies have focused on the bacterial and archaeal microbial communities (Hess et al., 2011; Pope et al., 2012; Wang et al., 2013; Kamke et al., 2016; Pitta et al., 2016) and lack targeted analysis of eukaryotic genes. Only a few of the mentioned studies detected eukaryotic genes at a low level (Hess et al., 2011; Wang et al., 2013; Pitta et al., 2016). In addition, Brulc et al. (2009) analyzed eukaryotic SSU genes and environmental gene tags (EGTs) from metagenomics data derived from fibrous and non-fibrous rumen samples. No fungal sequences were identified by phylogenetic analysis, but 19% of the detected eukaryotic EGTs were assigned to fungi, not comprising anaerobic fungi expected in the sampled environment. These results are confounding, as they depict rumen eukaryotes as a negligible group in the rumen microbial ecosystem. However, the absence of these eukaryotes may not have been responsible for these findings.

The lack of eukaryotic genes detected in metagenomics studies to date are likely to be caused by sampling strategies excluding eukaryotes, the low eukaryotic DNA content in the rumen (relative to their microbial biomass) and their scarce genetic information, limiting bioinformatics analysis and annotation of eukaryotic genes (Qi et al., 2011). Activity based screening of a dairy cow metagenome library cloned in *Escherichia coli*, in which anaerobic fungi accounted for 5% of the identified coding sequences, showed that if suitable methods were applied all existing rumen microbial groups were detected (Ferrer et al., 2005).

From a rumen perspective, there is a need to link the available anaerobic fungal genomic data with ecology and function and thus build a more comprehensive database. On this basis, bioinformatics approaches able to identify and annotate anaerobic fungal genes can be developed, enabling scientists to screen rumen metagenome data sets for anaerobic fungal gene content. This will prevent these important fiber degraders from being overlooked in future studies. Furthermore, sampling strategies for future rumen metagenomics studies need to be adjusted to anaerobic fungal cell size (e.g., large enough pore sizes when using nylon bags), growth characteristics (e.g., their growth inside plant fibers as rumen fluid only contains their zoospores) and behavior during nucleic acid extractions (see sections Requirements for culturing and genomic DNA isolation and Future perspectives).

## Transcriptomics

Whilst genome-based analysis gives insight into the fundamental biology of anaerobic fungi, gene expression brings our understanding a step closer to their actual activity and metabolism. In practical terms, it also overcomes the issue of eukaryotic genomes containing non-coding introns within their genes. Analysis of expressed anaerobic fungal genes, however, is not new, with the first work in this area being conducted more than 20 years ago, and having already generated fundamental biological insights on a limited subset of genes.

Reymond et al. (1992) determined an anaerobic fungal cDNA sequence, the phosphoenolpyruvate carboxykinase-encoding gene from *Neocallimastix frontalis*. Analysis of the gene's predicted protein structure revealed that the catalytic regions were highly conserved among anaerobic fungal and animal organisms, however, the yeast sequence showed no similarity to the *N. frontalis* sequence. Gilbert et al. (1992) isolated and characterized a xylanase cDNA from the rumen anaerobic fungus *Neocallimastix patriciarum*. Sequence analysis demonstrated significant homology between this enzyme and bacterial xylanases, which implied the horizontal transfer of genes between bacteria and anaerobic fungi in the rumen. Subsequently, this research group established a *N. patriciarum* cDNA library that was screened for xylanases (Xue et al., 1992), and they then modified the xylanase cDNA to obtain a high-level expression of the enzyme in *E. coli* (Xue et al., 1995).

Whilst other cDNA library based studies have also been conducted, most have focused on a limited number of hydrolytic enzymes (Supplementary Table 1). The first of the more high-throughput studies was conducted by Kwon et al. (2009), where they constructed an expressed sequence tag (EST) library of the rumen fungus *N. frontalis*. The functional genes from the library were analyzed to elucidate the carbohydrate metabolism pathways of this anaerobic fungus. With the development of next-generation sequencing technology, however, transcriptome based analysis has become the method of choice. Transcriptomics can be either the study of global gene expression as a function of different conditions (i.e., RNAseq) or the generation of a transcriptome for *de novo* assembly/annotation (often in combination with genome sequencing). In this section, we review the recent transcriptomic developments that have occurred with anaerobic fungi.

### Gene expression analysis of axenic/monoxenic anaerobic fungal cultures

The transcriptomes of four anaerobic fungi (*P. ruminantium* C1A, *Piromyces finnis, Neocallimastix californiae*, and *Anaeromyces robustus*) have been published to date (Couger et al., 2015; Solomon et al., 2016a), with more currently in progress within the wider research community. All the published transcriptomes show that anaerobic fungi produce several types of lignocellulolytic enzymes including glycosyl hydrolases (GH), polysaccharide lyases (PL) and carboxyl esterases (CE). *Pecoramyces ruminantium* C1A for example produced 44 GH families (385 transcripts), 8 PL families (43 transcripts), and 14 CE families (252 transcripts) (Couger et al., 2015). Multiple functionally redundant CAZymes were expressed, which were suggested to improve the speed and extent of plant biomass degradation. Comparison of anaerobic fungi and aerobic fungi also revealed much more biomass degrading genes were present in anaerobic fungi, especially cellulase (GH1, GH5, GH8, GH9, GH45, GH48) and hemicellulase (GH10, GH11) genes (Solomon et al., 2016a). In fact, anaerobic fungi contain the highest number of biomass degrading genes of any sequenced microbe on Earth (to date). This further highlights why anaerobic fungi are currently of such intense interest with respect to their carbohydrate active enzymes.

Whilst the statistics of the transcriptome assemblies suggest that many transcripts are present (Table 3), it was shown with *P. ruminantium* C1A that less than half of the total transcripts detected were expressed when the fungus was cultivated. This was irrespective of which substrate was used: glucose (32.3% of total transcripts), alfalfa (28.6%), energy cane (30.2%), sorghum (29.1%), and corn stover (27.0%) (Couger et al., 2015). This suggests that there is a limited proportion of core genes expressed when different substrates are utilized. As with the genomes, however, much of the transcripts in anaerobic fungi cannot be functionally annotated by any database, which causes a significant hurdle when interpreting the data.

**Table 3:** Statistics of *de novo* assembled transcripts of anaerobic fungi.

**Strains**	**Substrates**	**Transcripts (#)**	**Length (bp)**	**Length max (bp)**	**Length min (bp)**	**Average length (bp)**
*Anaeromyces robustus^a^*	Glucose, reed canary grass, Avicel, cellobiose, filter paper	17,127	21,955,935	21,526	100	1,281.9
*Neocallimastix californiae*^a^	Glucose, reed canary grass, Avicel, cellobiose, filter paper	29,649	36,250,970	19,022	100	1,222.7
*Pecoramyces ruminantium* C1A^b^	Glucose, alfalfa, energy cane, corn stover, sorghum	35,126	33,569,440	14,646	301	955.7
*Piromyces finnis*^a^	Glucose, reed canary grass	27,140	25,770,853	18,057	201	949.6

Data from

a Solomon et al. (2016a) and

b *Couger et al. (2015)*.

It has been reported that only 47.2% of all of the *A. robustus* transcripts could be annotated by NCBI BLAST, InterProScan, and OrthoMCL alignments (Solomon et al., 2016a). Analysis of the 10,639 transcripts present in *P. ruminantium* C1A grown with all substrates (glucose, alfalfa, energy cane, corn stover, and sorghum) indicates that functional annotation with KEGG (2,755 annotated, 25.8%), TrEMBL (2,972 annotated, 27.9%) and SwissProt (1,046 annotated, 9.8%) databases is even more limited for this fungus. In all three databases, 894 transcripts were annotated, only 15 of which were highly expressed (normalized FPKM > 1,000) with all substrates. Despite using a combination of the three databases, 7,524 transcripts were not annotated at all. This is even more alarming when it is considered that 87 of these non-annotated transcripts were highly expressed with all substrates.

**Figure 5:**
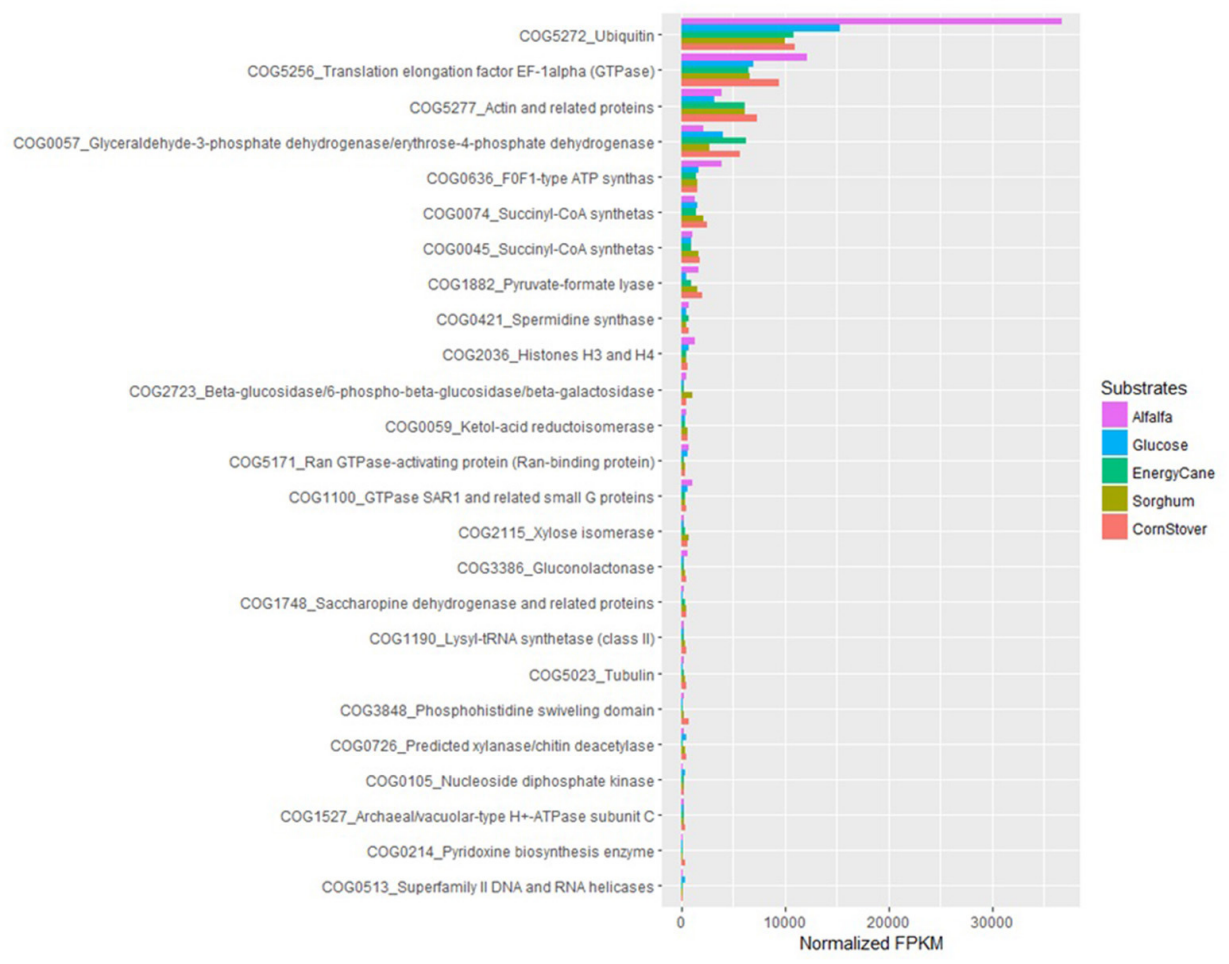
COG analysis of the core protein encoding transcripts that were present in *Pecoramyces ruminantium* C1A grown in five different substrates (glucose, alfalfa, energy cane, corn stover, and sorghum). Transcripts with a normalized FPKM above 100 are presented. Data sourced from Couger et al. (2015).

With COG ontology analysis 1,461 transcripts were annotated from *P. ruminantium* C1A, 18 of which were highly expressed with all substrates (normalized FPKM > 1,000). Within the 9,178 transcripts not annotated by COG, 92 were highly expressed with all substrates (normalized FPKM > 1,000). Interestingly, the 87 transcripts (which could not be functionally annotated by KEGG, TREMBL or SwissProt) are all included in these 92 core transcripts which could not be annotated by COG. Among the 18 COG annotated core transcripts, ribosomal RNA dominated (Log (normalized FPKM) values ranging from 3.21 to 5.18), followed by proteins (Figure 5) involved in metabolic pathways in the cytosol and hydrogenosome, and plant fiber degradation. As the unannotated highly expressed core transcripts are likely to play a central role in anaerobic fungal metabolism, it is important that future studies are performed in order to determine their function.

Couger et al. (2015) reported that the transcriptional levels of the majority of CAZyme families and transcripts in *P. ruminantium* C1A were not significantly altered by complex substrates. This is in contrast to the study of Solomon et al. (2016a), who reported that the expression of CAZymes in *P. finnis* was repressed in the presence of glucose, and induced during growth on more complex, insoluble substrates such as filter paper, Avicel, and reed canary grass (*Phalaris arundiacea*). It is not clear, however, to what extent the differences in these analyses may be attributed directly to differences in the underlying biology of diverse strains of anaerobic fungi, or due to differences in the technical approaches employed. Data for *P. ruminantium* was averaged from only two replicates, without indicating associated error in FPKM levels, while data for *P. finnis* from Solomon et al. (2016a) utilized three biological replicates and presented the standard error of the mean for all data. Expression levels across biological replicates can vary widely, and many RNA-Seq statistical analysis packages rely on incorporation of sufficient biological replicates for accurate results (Tarazona et al., 2011). For example, Schurch et al. (2016) recommends that more than 3 replicates per condition are required for confidence in a log_2_-fold change ≤2.0 when using the DESeq analysis package. With ongoing reduction in costs of Next Generation Sequencing, it is critical to incorporate appropriate biological replicates and report error associated with expression measurements to improve confidence in results from differential expression analyses. Differences in the repression/induction of CAZyme gene expression between anaerobic fungal genera is, however, perhaps not so surprising, and suggests that the concept of niche differentiation within anaerobic fungi (Griffith et al., 2009) may be related to differences such as these.

### Metatranscriptomics

Utilizing metatranscriptomics for the study of eukaryotic derived activity in mixed communities has many advantages (Marmeisse et al., 2017); particularly due to the syntrophic interactions that occur between anaerobic fungi and other rumen microbes (see section Metabolomics). Recently, a number of studies have used metatranscriptomics to examine the rumen microbial community (Qi et al., 2011; Wang et al., 2011; Poulsen et al., 2013; Dai et al., 2014; Kamke et al., 2016; Li F., et al., 2016; Comtet-Marre et al., 2017; Hinsu et al., 2017; Li and Guan, 2017). However, very few of these studies identified significant numbers of anaerobic fungal transcripts in their results. Although Dai et al. (2014) found significant numbers of anaerobic fungal carbohydrate binding domains (CBM10) associated with GH48 cellulases, the anaerobic fungi were only able to be associated with less than 1% of the total reads.

In contrast, Qi et al. (2011) used a polyadenylated RNA capture technique that specifically enhanced the sequencing of eukaryotic transcripts. This resulted in the characterization of a large number of rumen anaerobic fungal enzymes associated with cell wall degradation. This included enhanced representation of GH6 and GH48 cellulases, that are virtually absent from rumen metagenomes, and large numbers of CBM associated enzymes. Qi et al. (2011) also had an 8.7x higher discovery rate of CAZymes compared to previous metagenomics studies.

Qi et al. (2011) noted that many of the discovered CAZymes were most closely related to rumen bacterial sequences, consistent with other reports that gene transfer has taken place (Gilbert et al., 1992; Haitjema et al., 2017). Rumen fungal sequences represented 14.4% of the total identified reads; however 63.8% of the reads were unable to be classified. This clearly demonstrates the limitations of transcriptomic analysis when databases used for annotation do not contain significant characterized sequences of anaerobic fungi. Also, it may be possible that many anaerobic fungal CAZymes may be attributed to being bacterially produced in a ruminal sample when they have actually been expressed by an anaerobic fungus.

Recently, three studies used sequencing of total RNA, not depleted of rRNA, to examine the active microbial community in the rumen (Poulsen et al., 2013; Li F., et al., 2016; Elekwachi et al., 2017). However, only the study of Elekwachi et al. (2017) found significant contributions of anaerobic fungi. Elekwachi et al. (2017) found between 10 and 16% of the total rRNA reads were of anaerobic fungal origin, with the genera mainly consisting of *Neocallimastix* (56%), *Cyllamyces* (36%), and *Orpinomyces* (8%). The main differences between the study of Elekwachi et al. (2017) and the studies that do not identify many anaerobic fungal sequences can be attributed to differences in animal diet and sample preparation (section Future Perspectives).

Rumen anaerobic fungi are intimately associated with the fiber portion of the diet, with their main active vegetative growth occurring within the plant cell wall matrix. This makes sample preparation of utmost importance if rumen fungal sequences are to be discovered. Sampling and preparation methods that do not contain a representative quantity of rumen fiber and that do not aggressively disrupt the plant cell wall matrix (or the anaerobic fungus itself, see section Requirements for culturing and genomic DNA isolation) are unlikely to be successful in obtaining a truly representative sample of the rumen microbial community and have significant bias against anaerobic fungi (Wang et al., 2011).

## Proteomics

Proteomics bridges the gap between transcriptomics and metabolomics, and permits the large scale analysis of proteins. Proteomics can be classified as being either native or translated proteome analysis. The latter involves the translation of individual mRNAs or transcriptomes prior to the proteome analysis, and has been made possible due to the development of NGS techniques. Translated proteome analysis, however, cannot assess post-translational modifications such as glycosylation or methylation, which requires more targeted analyses.

**Figure 6:**
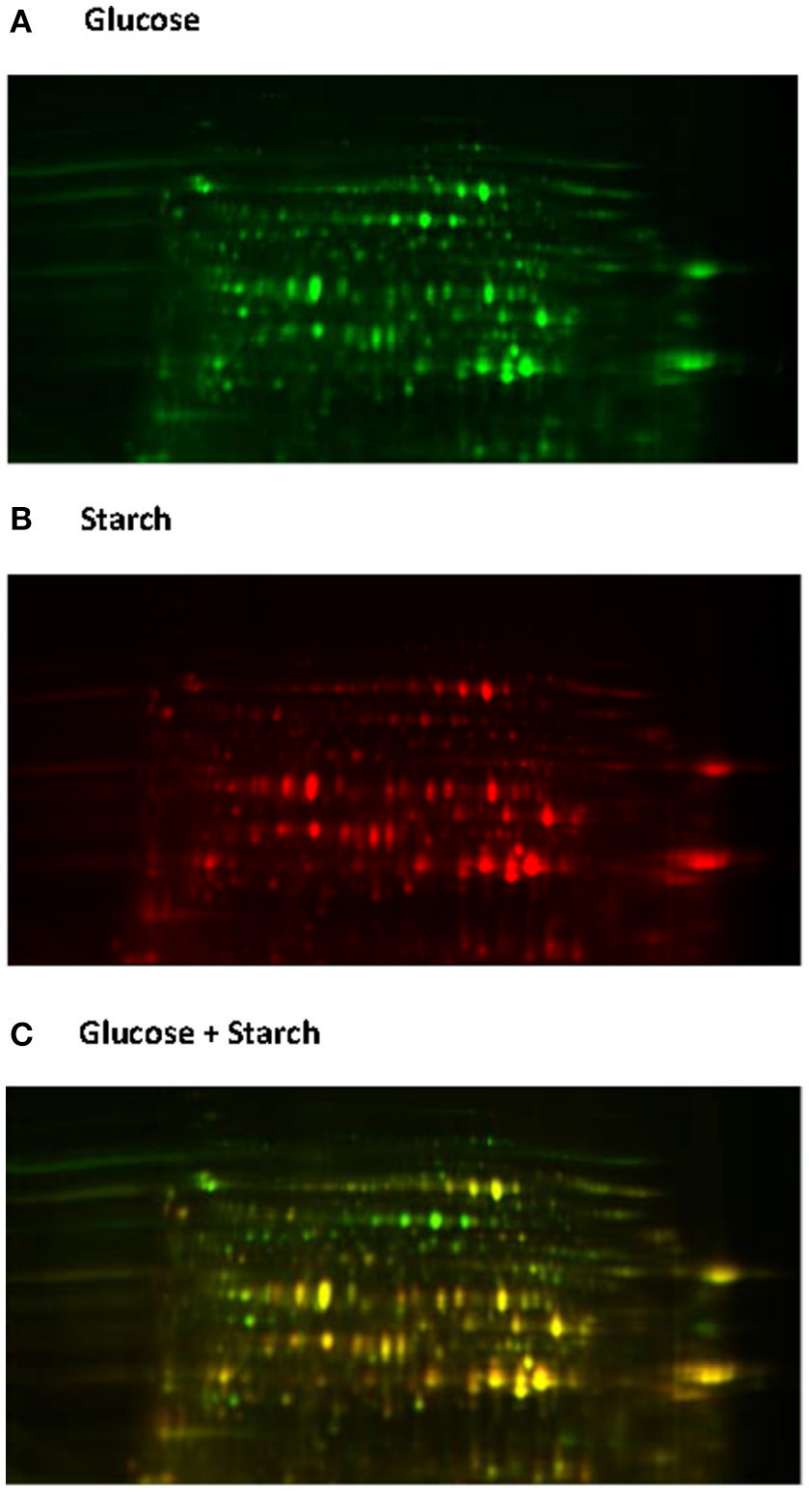
2D-DIGE analysis of the *Neocallimastix frontalis* proteome. Images of *N. frontalis* cultures grown on glucose **(A)** and starch **(B)** are shown in the Cy3 and Cy5 channels, respectively, as well an image of the combined Cy3 + Cy5 channels **(C)**.

Native proteome analysis involves three key steps: protein separation, sequence analysis and protein identification. The current standard for protein separation utilizes liquid chromatography (LC), where proteins are separated based on characteristics such as polarity and molecular weight (Lin et al., 2003), and then directly analyzed using mass spectrometry (MS). Due to technical advances in MS and the laboriousness of other methods, direct proteome analysis with LC-MS is now becoming the method of choice (Feist and Hummon, 2015). However, only two studies to date have utilized such methods for analyzing anaerobic fungi (Solomon et al., 2016a; Haitjema et al., 2017). Protein separation can also be performed using gel electrophoresis, either one or two dimensional gel electrophoresis (2D-GE). With 2D-GE, native proteins are separated based on isoelectric point (pI, the horizontal first dimension) and molecular weight (the vertical second dimension) (Gorg et al., 2004). The 2D-GE can provide information about individual proteins within a gel; however, due to the variations in protein separation among 2D gels it can be difficult to compare protein expression between biological samples. This has led to the development of two dimensional differential gel electrophoresis (2D-DIGE) (Gorg et al., 2004). With 2D-DIGE protein samples are pre-stained with different fluorescent dyes, and then run on the same gel in order to eliminate gel to gel variation (Figure 6). Sequencing of the gel separated proteins, however, requires additional steps to excise the individual proteins, and then sequence them either with Edman degradation (Aebersord et al., 1987) or mass spectrometry (MS) based methods after digestion (Graham et al., 2007).

Protein identification is performed by searching the acquired peptide mass results of each protein against a database, with the protein database being the key factor for success in identifying a protein. Within the 452 protein sequences currently available in the NCBI database (representing less than 0.3% of the deposited rumen bacterial protein sequences) only four filamentous anaerobic fungal genera are currently represented. Furthermore, the deposited proteins are also heavily biased toward CAZymes due to these being a primary area of research focus. Of the 452 protein sequences there are 109 hexose degrading enzymes and 46 pentose degrading enzyme, whilst only 102 of the deposited sequences are related with intra-cellular carbohydrate metabolism including glycolysis, hydrogenosome metabolism and pseudo-TCA cycle (Kwon et al., 2009).

**Figure 7:**
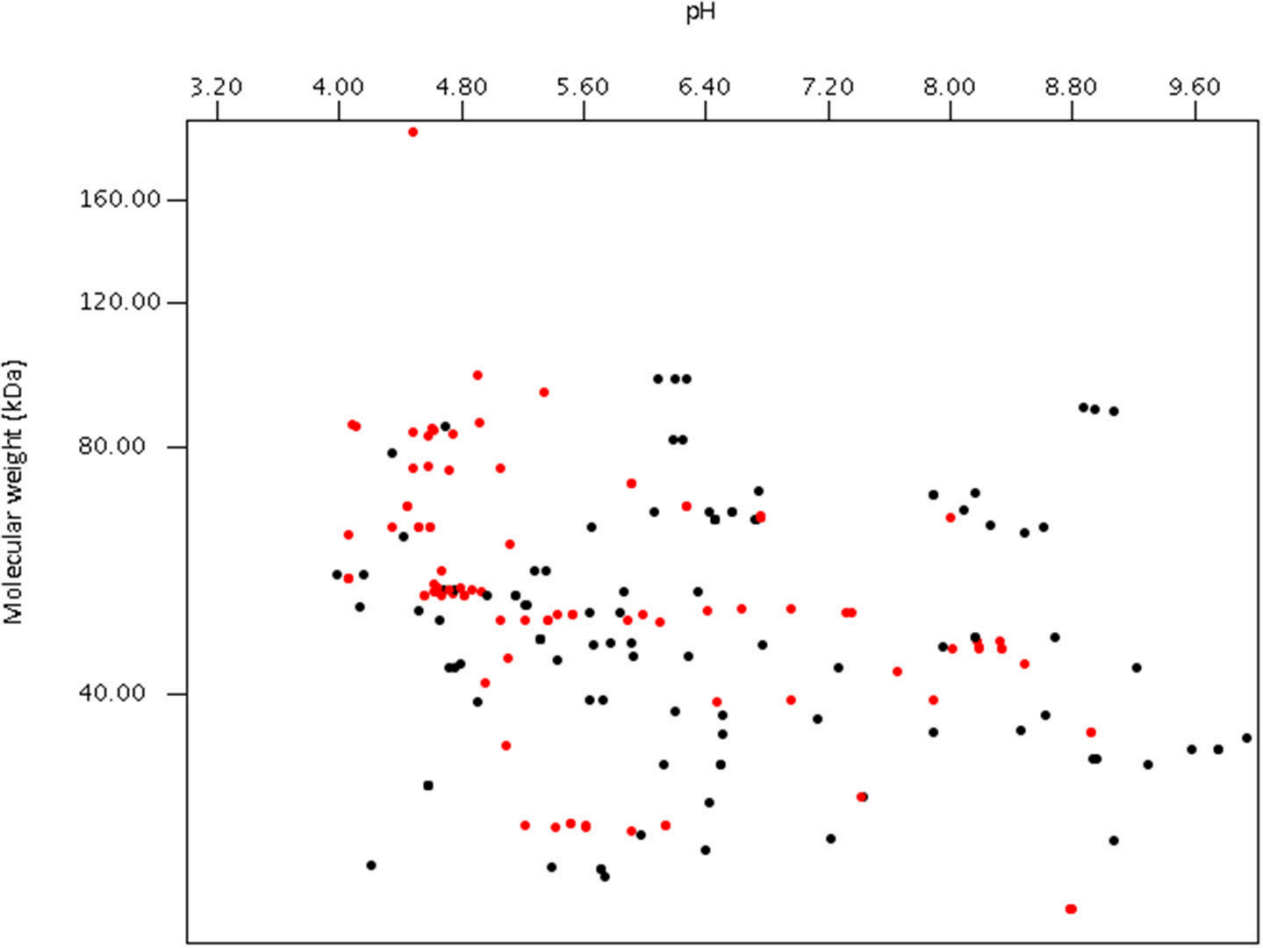
Virtual 2D-gel of anaerobic fungal proteins using data obtained from the NCBI database (203 protein sequences) and generated using JVirGl 2.2.3b software (www.jvirgel.de). Predicted secretory proteins (Supplementary Table 2) are shown as red dots, and non-secretory proteins (Supplementary Table 3) as black dots.

Using publically available protein data, a virtual proteome map was constructed (Figure 7). Among the 203 protein sequences analyzed, 97 (including cellulase, xylanase, lichenase, and acetylxylan esterase) were predicted to be secretory proteins and the other 106 non-secretory. Of the 106 non-secretory intracellular proteins, 29 were related with hexose or pentose degrading enzymes. The average molecular size of the 203 deposited proteins was 50 kDa. The largest protein was endoglucanase 5A (CAB92326.1) from *Piromyces equi* (Eberhardt et al., 2000) with 192.9 kDa, and it contained four GH5s and CBM10s. This enzyme was the only reported anaerobic fungal protein bigger than 100 kDa, and was predicted to be secretory. In contrast, cyclophilin B and its precursor, also predicted to be secretory, were the smallest protein at 19.7 kDa (Chen et al., 1995). More recent proteomic analyses have shown that the size of proteins detected from anaerobic fungi ranges much more widely than previously determined (Haitjema et al., 2017). For instance, a 694.2 kDa protein was observed in the secretome using LC-MS methods, which was not detected using standard gel-based separations due to its large size.

In addition to more advanced proteomics techniques to reach a wider range of proteins, targeted proteomics are required to assess the post-translational modifications previously shown to decorate some of the anaerobic fungal proteins (Haitjema et al., 2017). These targeted methods most commonly consist of a preliminary capture step, where proteins bearing the desired modification are captured using a specific antibody or sugar-binding lectin in the case of glycosylation. The captured proteins are then analyzed using MS. Additionally, glycosylation patterns can be analyzed by treating proteins with a deglycosylation mix and then observing the released sugars with MS. Along with these sample preparation considerations, protein identification is also a challenge in anaerobic fungal proteomics studies. For example, Park (2011) reported that of the 800 protein spots that were selected from gels and analyzed using MS, only 180 protein sequences were identified due to database limitations. The current challenges facing the research community, therefore, are to improve sample preparation procedures as well as increase the amount of available annotated sequence data.

## Metabolomics

Metabolomics refers to the qualitative and quantitative analysis of the metabolites present inside (endometabolome) and outside (exometabolome) growing cells at a given time (Mashego et al., 2007). The metabolome of an organism is comprised of numerous different types of metabolites (e.g., carbohydrates, fatty acids, alcohols, organic acids, amino and non-amino acids, lipids, etc.) with each having a different origin and function (Villas-Bôas et al., 2005). As the metabolites define the phenotype of an organism, their analysis gives insights into both genetic and environmental changes. Furthermore, metabolomics helps to provide less biased information on genotype and phenotype (Abdelnur et al., 2014), as information contained in the genome, transcriptome or proteome does not always result in the phenotype. Metabolomics, therefore, is a complementary method to use in combination with other ‘omics techniques (Villas-Bôas et al., 2005; Mashego et al., 2007). Despite several technical advancements in sample processing and data analysis, however, no single technique can fully elucidate the metabolome. Therefore, a combination of different approaches is required, with mass spectrometry (MS) and nuclear magnetic resonance (NMR) currently being the most commonly used.

Three distinct approaches are used in metabolomics studies: targeted analysis, metabolite profiling and metabolic fingerprinting. Each of these approaches however, has its own advantages and disadvantages (Shulaev, 2006). Targeted analysis is the most developed quantitative approach, whereas metabolite fingerprinting and metabolite profiling are more global approaches that are only semi-quantitative. Numerous studies have been published on the targeted analysis of anaerobic fungal metabolite profiles of several genera in axenic culture (Table 4). In contrast, only one non-targeted metabolomics based study of anaerobic fungi has been published to date (Cheng et al., 2013).

**Table 4:** Fermentation end products of anaerobic fungal metabolism of different substrates.

**Anaerobic fungal culture**			**Fermentation products^b^^,^^c^^,^^d^**									
**Genus**	**Species or strain^a^**	**Substrate**	**H_2_**	**CO_2_**	**F**	**A**	**Lactate**			**S**	**E**	**References**
							**DL**	**LL**	**TL**			
*Neocallimastix*	*patriciarum*	Cellulose	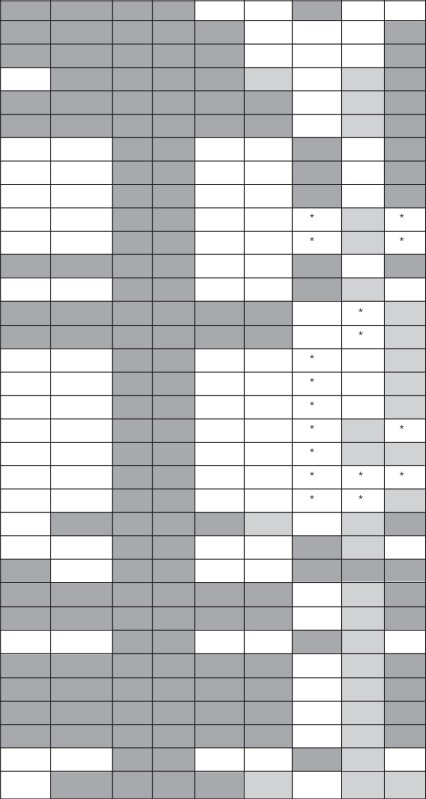									Orpin and Munn, 1986
	*hurleyensis*	Glucose										Lowe et al., 1987
		Xylose										
	LM1	Glucose										Phillips and Gordon, 1988
	MC2	Coastal bermuda grass										Borneman et al., 1989
		Glucose										
	NC71	Wheat straw										Kostyukovsky et al., 1991
		Filter paper										
		Cellobiose										
	*variabilis*	Rice straw										Ho et al., 1996
		Filter paper										
	*frontalis*	Cellulose										Srinivasan et al., 2001
	spp.	Glucose										Paul et al., 2010
*Piromyces*	MC1	Coastal bermuda grass										Borneman et al., 1989
		Glucose										
	PC12	Wheat straw										Kostyukovsky et al., 1991
		Filter paper										
		Cellobiose										
	*spiralis*	Rice straw										Ho et al., 1996
		Filter paper										
	*minutus*	Rice straw										
		Filter paper										
	SM1	Glucose										Phillips and Gordon, 1988
	spp.	Glucose										Paul et al., 2010
	E2	Fructose										Boxma et al., 2004
*Anaeromyces*	PC1	Coastal bermuda grass										Borneman et al., 1989
		Glucose										
	spp.	Glucose										Paul et al., 2010
*Orpinomyces*	PC2	Coastal bermuda grass										Borneman et al., 1989
		Glucose										
	PC3	Coastal bermuda grass										
		Glucose										
	spp.	Glucose										Paul et al., 2010
*Caecomyces*	NM1	Glucose										Phillips and Gordon, 1988

a *Species or strain names are given as described in the corresponding publication, with “spp.” indicating that the same results were found with the multiple strains that were tested in a given study*.

b *Fermentation products: hydrogen (H_2_), carbon dioxide (CO_2_), formate (F), acetate (A), succinate (S), ethanol (E), D(–) lactate (DL), L(+) lactate (LL), and total lactate (TL) (in publications where the specific isomers were not reported)*.

c Fermentation products were produced (dark gray), produced in low or trace amounts (light gray), assessed but not detected (^*^) or were not assessed (empty cells).

d *Hydrogen and carbon dioxide are always produced by anaerobic fungi. If they are not mentioned in the table, it means they were not estimated or their details were not given in the associated reference*.

The relative abundances of the metabolites produced by anaerobic fungi are likely to vary based on substrate or strain. The major fermentation products of all species of anaerobic fungi in axenic culture, however, are hydrogen, carbon dioxide, formate, acetate and lactate, with succinate, and ethanol normally produced in lesser quantities (Table 4). Propionate and butyrate, major fermentation products of rumen bacterial metabolism, are not produced by anaerobic fungi. Using an NMR-based metabolomic approach, Cheng et al. (2013) showed for the first time that α-ketoglutarate was also a major fermentation product of anaerobic fungi. Lactate, succinate, α-ketoglutarate, and ethanol are produced in the cytosol of anaerobic fungi, while hydrogen, carbon dioxide, and acetate are produced in the hydrogenosome (O'Fallon et al., 1991; Kwon et al., 2009; Cheng et al., 2013). Formate is the only product that is produced in both the cytosol as well as the hydrogenosome. In addition to major fermentation products, many fungi produce molecules termed secondary metabolites, which have putative functions in diverse roles such as defense, signaling, and stress response (Keller et al., 2005). Secondary metabolites are an important class of molecule to study, because they have been harvested from other clades of fungi for use as antimicrobial compounds, anti-tumor agents, and insecticides. They are often difficult to characterize, because they are typically produced at low levels or only under certain conditions. To date, there have been no studies characterizing the secondary metabolites produced by anaerobic fungi.

Numerous *in vitro* studies have shown that some of the typical end products of anaerobic fungal metabolism are often not accumulated when the fungi are grown in co-culture (Table 5). Hydrogenotrophic methanogens utilize hydrogen and formate as substrates, resulting in the production of methane (Theodorou et al., 1996). In contrast, co-culturing of anaerobic fungi with the aceticlastic methanogen *Methanosarcina barkeri* resulted in the accumulation of formate, and conversion of acetate into methane (Mountfort et al., 1982). Aceticlastic methanogens however represent a very minor part of the rumen archaeal community (Henderson et al., 2015). Numerous studies have confirmed increased production of carbon dioxide and acetate in the presence of hydrogenotrophic methanogens, while the production of lactate, succinate, α-ketoglutarate, and ethanol decreases (Table 5). Cheng et al. (2013) also showed that citrate was a major fermentation product of anaerobic fungi, when these were grown in co-culture with methanogens.

**Table 5:** Changes in fermentation end products of anaerobic fungal metabolism in axenic culture compared to co-culture with methanogens, bacteria or protozoa.

**Anaerobic fungus**	**Coculture partner**	**Substrate**	**Fermentation products^a^^,^^b^**		**Significant changes in fermentation patterns due to cocultivation^c^**	**References**
			**Fungal monoculture**	**Fungal co-culture**		
**ANAEROBIC FUNGI WITH METHANOGENS**						
*Neocallimastix frontalis* PN1	*Methanobrevibacter* sp. strain RA1	Cellulose	H_2_, CO_2_, F, A, L, E	CO_2_, A, L, E, CH_4_	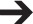 of F & H_2_ into CH_4_; 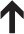 A, CO_2;_ 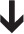 L, E	Bauchop and Mountfort, 1981
	*Methanosarcina barkeri*			CO_2_, F, A, L, E, CH_4_	Initial 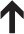 in H_2_ & A followed by 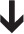	Mountfort et al., 1982
	*Methanosarcina barkeri* & *Methanobrevibacter* sp. strain RA1			CO_2_, L, E, CH_4_	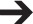 of F & A into CH_4_; 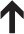 CO_2_, CH_4_; 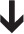 L, E	
*Neocallimastix frontalis* RE1	*Methanobacterium arboriphilus*	Filter paper	H_2_, CO_2_, F, A, L, S, E	CO_2_, F, A, S, E, CH_4_	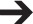 of H_2_ into CH_4_; 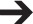 or partial 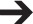 of F into CH_4_; 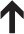 A; 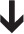 S, L, E; 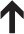 CO_2_ only with *Methanobrevibacter smithii*	Marvin-Sikkema et al., 1990
	*Methanobacterium bryantii*					
	*Methanobrevibacter smithii*			CO_2_, A, S, CH_4_		
*Neocallimastix patriciarum* CX	*Methanobacterium arboriphilus*			CO_2_, F, A, S, E, CH_4_		
	*Methanobacterium bryantii*					
	*Methanobrevibacter smithii*			CO_2_, A, S, E, CH_4_		
*Neocallimastix* sp. L2	*Methanobacterium arboriphilus*			CO_2_, F, A, E, CH_4_		
	*Methanobacterium bryantii*			CO_2_, F, A, S, E, CH_4_		
	*Methanobrevibacter smithii*			CO_2_, A, S, E, CH_4_		
*Neocallimastix frontalis*	*Methanobacterium formicicum*	Cellulose	H_2_, F, A, L, E	A, L, E, CH_4_	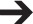 of F & H_2_ into CH_4_; 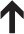 A; 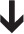 L, E	Nakashimada et al., 2000
	*Methanosaeta concilii*			H_2_, F, A, L, E, CH_4_	Partial 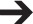 of F & H_2_ into CH_4_; 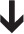 H_2_, F, A, L, E	
	*Methanobacterium formicicum* & *Methanosaeta concilii*			A, L, E, CH_4_	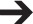 of F & H_2_ into CH_4_; L, E	
*Neocallimastix frontalis* Yaktz1	*Methanobrevibacter ruminantium*	Wheat straw	F, A, L, E	A, L, CH_4_, E	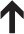 A; 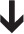 L, E	Wei et al., 2016b
		Corn stalk				
		Rice straw				
*Piromyces communis* P	*Methanobacterium arboriphilus*	Filter paper	H_2_, CO_2_, F, A, L, S, E	CO_2_, F, A, S, E, CH_4_	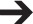 of H_2_ into CH_4_; 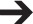 or partial 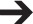 of F into CH_4_; 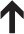 A; 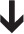 S, L, E; 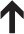 CO_2_ only with *Methanobrevibacter smithii*	Marvin-Sikkema et al., 1990
	*Methanobacterium bryantii*			CO_2_, F, A, E, CH_4_		
	*Methanobrevibacter smithii*			CO_2_, A, S, E, CH_4_		
*Piromyces communis* FL	*Methanobrevibacter ruminantium*		H_2_, F, A, L, E	A, L, E, CH_4_	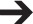 of F & H_2_ into CH_4_; 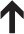 A; 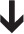 L, E	Bernalier et al., 1992
*Piromyces* sp.	*Methanobrevibacter thaueri*	Corncob	F, A, L, E	A, L, CH_4_	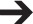 of F into CH_4_; 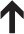 A	Jin et al., 2011
*Piromyces* sp. F1		Cellobiose	F, A, L, S, C, α-K, E	F, A, L, S, C, α-K, E	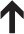 F, A, L, S, C, E; 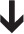 α-K	Cheng et al., 2013
Mixed coculture of anaerobic fungi & methanogens derived from goat rumen						
*Caecomyces communis* FG10	*Methanobacterium arboriphilus*	Filter paper	H_2_, CO_2_, F, A, L, S, E	CO_2_, F, A, E, CH_4_	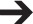 of H_2_ into CH_4_; 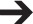 or partial 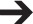 of F into CH_4_; 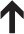 A; 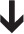 S, L, E; 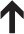 CO_2_ only with *Methanobrevibacter smithii*	Marvin-Sikkema et al., 1990
	*Methanobacterium bryantii*			CO_2_, F, A, S, E, CH_4_		
	*Methanobrevibacter smithii*			CO_2_, A, S, L, E, CH_4_		
	*Methanobrevibacter ruminantium*		H_2_, F, A, L	A, L, CH_4_	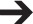 of F & H_2_ into CH_4_; 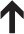 A; 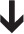 L	Bernalier et al., 1991
**ANAEROBIC FUNGI WITH BACTERIA**						
*Neocallimastix frontalis* MCH3	*Ruminococcus flavefaciens*	Filter paper	H_2_, CO_2_, F, A, L, E	H_2_, CO_2_, F, A, L, E	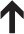 CO_2_, A; 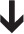 H_2_, F, L, E	Bernalier et al., 1992
	*Fibrobacter succinogenes*				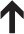 CO_2;_ 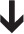 H_2_, F, A, L, E	
	*Eubacterium limosum*		H_2_, CO_2_, F, A, L, E	H_2_, CO_2_, A, B, L, E	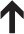 CO_2_, A_;_ 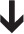 H_2_, L, E	Bernalier et al., 1993
*Piromyces communis* FL	*Selenomonas ruminantium*		H_2_, F, A, L, E	H_2_, A, P, L, E	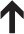 A, L, E; 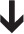 H_2_	Bernalier et al., 1991
	*Ruminococcus flavefaciens*		H_2_, CO_2_, F, A, L, E	H_2_, CO_2_, F, A, L, E	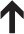 CO_2_, A; 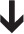 H_2_, F, L, E	Bernalier et al., 1992
	*Fibrobacter succinogenes*				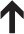 CO_2;_ 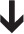 H_2_, F, A, L, E	
	*Eubacterium limosum*		H_2_, CO_2_, F, A, L, E	H_2_, CO_2_, A, B, L, E	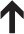 CO_2_, A_;_ 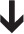 H_2_, L, E	Bernalier et al., 1993
	*Ruminococcus flavefaciens*	Maize Stem	H_2_, F, A, L, E	H_2_, F, A, L, E	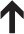 F, A; 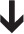 E	Roger et al., 1992
	*Fibrobacter succinogenes*					
*Piromyces communis* B19	*Ruminococcus flavefaciens*	Xylan	F, A, L, S	F, A, L, S	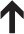 S; 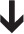 A	Williams et al., 1994
	*Butyrivibrio fibrisolvens*			F, L	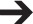 of A; 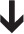 L	
	*Prevotella ruminicola*			F, A, L, S	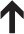 A, S; 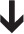 F, L	
	*Succinivibrio dextrinosolvens*			F, A, S	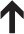 A, S; 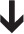 F	
	*Streptococcus bovis*			F, A, L, S	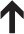 L; 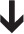 A	
	*Veillonella parvula*			F, A, L	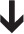 A, L	
*Caecomyces communis* FG10	*Selenomonas ruminantium*	Filter paper	H_2_, F, A, L	H_2_, A, P, L, E	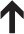 E; 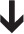 H_2_, A	Bernalier et al., 1991
	*Ruminococcus flavefaciens*		H_2_, CO_2_, F, A, L, E	H_2_, CO_2_, F, A, L, E	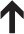 CO_2_, L; 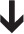 H_2_, F, A, E	Bernalier et al., 1992
	*Fibrobacter succinogenes*		H_2_, CO_2_, F, A, L, E	H_2_, CO_2_, F, A, E	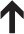 CO_2_, A, E; 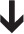 H_2_, F, L	
	*Eubacterium limosum*		H_2_, CO_2_, F, A, L, E	H_2_, CO_2_, A, B, L	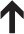 H_2_, CO_2_, A; 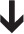 L	Bernalier et al., 1993
	*Ruminococcus flavefaciens*	Maize Stem	H_2_, F, A, L, E	H_2_, F, A, L, E	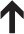 F, A; 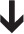 E	Roger et al., 1992
	*Fibrobacter succinogenes*					
**ANAEROBIC FUNGI WITH CILIATE PROTOZOA**						
*Neocallimastix patriciarum* CX	Ciliate protozoa	Rice straw	F, A, L	F, A, P, B	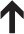 A; 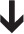 F; 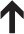 P & B at the expense of F & L	Widyastuti et al., 1995
*Piromyces* sp. strain OTS1	Mixed protozoa	Filter paper	F, A, L	A, P, B	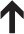 A; 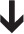 L	Morgavi et al., 1994a

a *Fermentation products: hydrogen (H_2_), carbon dioxide (CO_2_), formate (F), acetate (A), succinate (S), ethanol (E), lactate (L), citrate (C), α ketoglutarate (α-K) and methane (CH_4_)*.

b *Hydrogen and carbon dioxide are always produced by anaerobic fungi in monoculture. If they are not mentioned in the table, it means they were not estimated or their details were not given in the associated reference*.

c *The changes indicated are a conversion (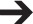), an increase (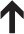) or a decrease (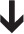)*.

As well as influencing metabolism, the presence of methanogens also enhances the lignocellulolytic activities of anaerobic fungi (Bauchop and Mountfort, 1981; Nakashimada et al., 2000; Leis et al., 2014). This is mainly due to interspecies hydrogen transfer leading to methane production and efficient regeneration of oxidized nucleotides like NAD+, NADP+ (Dollhofer et al., 2015). This syntrophic association of anaerobic fungi and methanogens is well defined, with methanogens also attaching themselves to fungal rhizoids and sporangia (Jin et al., 2011; Leis et al., 2014; Wei et al., 2016a). In contrast to the numerous methanogen co-culture studies though, a more limited number of studies with rumen bacteria and protozoa have been performed (Table 5).

Rumen fibrolytic bacteria and anaerobic fungi compete for the same substrates. However, the ability of anaerobic fungi to physically disrupt the plant particles using their invasive rhizoids gives them an advantage over bacteria when utilizing lignocellulosic substrates. The invasive growth of the anaerobic fungal rhizoids can, however, benefit the rumen bacteria by increasing surface area and/or access to internally exposed areas of the plant. Several studies have been conducted on cocultures of anaerobic fungi with fibrolytic and non fibrolytic bacteria (Table 5). The bacterial cultures generally result in a reduction of the fibrolytic activity of anaerobic fungi (Williams et al., 1991; Roger et al., 1992; Bernalier et al., 1993). Most of the fungal and bacterial co-culture studies have also shown increased carbon dioxide and decreased hydrogen, formate, lactate, and ethanol production relative to the fungal monoculture, with varying acetate levels (Table 5).

Rumen protozoa are generally thought to be antagonistic toward anaerobic fungi. Certain protozoa have been reported to prey on anaerobic fungal zoospores (Gordon and Phillips, 1998; Newbold et al., 2015), produce fungal cell wall degrading enzymes (Morgavi et al., 1994b) and decrease the fibrolytic activity of anaerobic fungi (Widyastuti et al., 1995). *In vivo*, it has also been shown that removal of protozoa from the rumen increases the anaerobic fungal population. Similar to fungal co-cultures with methanogens and bacteria, culturing of anaerobic fungi with protozoa also results in a fermentation shift from lactate toward enhanced acetate production (Table 5).

Overall, the information regarding anaerobic fungal metabolites is primarily from targeted analysis, and not all of the major metabolites have been measured in studies to date. Particularly, α-ketoglutarate and citrate production in axenic and co-cultures of anaerobic fungi respectively, which have only recently been discovered to be major products (Cheng et al., 2013). As a consequence there is a limited ability to systematically compare fermentation profiles of different anaerobic fungal genera/species/strains, especially in co-culture studies and when different substrates and/or media have been used. The novel insights gained by Cheng et al. (2013) also highlight that the use of global metabolomics approaches are key to furthering our understanding of the fundamental biology of anaerobic fungal metabolism both in pure and mixed culture.

## Future perspectives

Anaerobic fungi are the most effective fiber degrading organism in the herbivore gut, with numerous studies confirming their value as a direct fed microbial for ruminants. Reported benefits include improved feed intake, feed digestibility, feed efficiency, daily weight gain and milk production (Lee et al., 2000; Dey et al., 2004; Paul et al., 2004; Tripathi et al., 2007; Saxena et al., 2010; Puniya et al., 2015). Furthermore, anaerobic fungi have a well-established syntrophic interaction with rumen methanogens. On this basis, anaerobic fungi should therefore be of great interest in rumen microbial studies considering current research is focused on increasing the sustainability of ruminant livestock production and decreasing its environmental footprint. Despite this, the characterization of anaerobic fungi in rumen microbial ecology studies is not routinely undertaken, even though suitable molecular tools are available.

Molecular tools for ecological studies of anaerobic fungi now provide better depth of characterization and taxonomic resolution than before, as it is apparent that several of the previously used ITS1 primers did not provide complete coverage of the Neocallimastigomycota phylum. In addition to ITS1 based profiling methods such as ARISA and DGGE (Table 1), tools and data files have also been generated to support the bioinformatics analysis of NGS amplicon based analysis of this region (see section Internal transcribed spacer region). Several quantitative PCR methods also exist based on the ITS1 region (Denman and Mcsweeney, 2006; Lwin et al., 2011; Kittelmann et al., 2012; Marano et al., 2012), as well as the more highly conserved 5.8S rRNA gene (Edwards et al., 2008). The 28S rRNA gene, however, is likely to become the barcode of choice for targeted anaerobic fungal studies in the future due to its benefits over ITS1 as a barcoding locus for this phylum, although the combined use of both barcodes may also have merit. The ITS1, however, will remain of value in the detection of potential novel anaerobic fungal ITS1 sequences in wider environmental surveys, where the diversity of all fungi is of interest (Schoch et al., 2012). A recently published gene method for quantifying fibrolytic activity of cultures using cDNA also offers a new avenue to expand our understanding of how different factors affect fiber degradation by anaerobic fungi (Dollhofer et al., 2016). The increasing amount of anaerobic fungal genomic information will also in the future enable further such functional based quantification assays to be developed. This is important, as quantitatively anaerobic fungi are often incorrectly considered to be a minor part of the rumen microbial community.

A large proportion of early rumen microbial studies focused primarily on the analysis of rumen fluid, where anaerobic fungi only occur transiently as zoospores. Furthermore, whilst rumen zoospore numbers are low compared to counts of bacteria and archaea; anaerobic fungi have been reported to represent up to 20% of the rumen microbial biomass (Rezaeian et al., 2004): a figure comparable to the 10–16% of rRNA transcript abundance in metatranscriptomic studies (Elekwachi et al., 2017). This is because much of the rumen anaerobic fungal biomass is intimately associated with dietary plant material, due to the nature of the invasive growth of the rhizomycelium during the vegetative growth stage of the anaerobic fungal life cycle. This is why it is crucial that ruminal solids are also sampled when conducting rumen microbial studies, not just the ruminal liquid, as otherwise the amount of anaerobic fungi will be greatly underestimated. This is important not just for anaerobic fungi, however, as other microbial taxa also differ between ruminal liquid and solid fractions (Henderson et al., 2013).

Ruminal sampling is always best performed via a ruminal cannula, as oral stomach tubing is capable of sampling only small feed particles which are normally highly degraded. A ruminal sample of at least 500 grams should be obtained, ideally from the pooling of samples collected at multiple different locations (i.e., the front and middle of the ventral sac and the cranial sac). If required, this sample can then be portioned into liquid and fiber fractions using the French-press method described by Kong et al. (2010) or by using cheesecloth. The sample preparation method used to isolate RNA or DNA from the collected ruminal samples, however, will also have a large effect on whether or not the sample extract is truly representative in terms of anaerobic fungal nucleic acid content.

Subsamples of rumen content that utilize only 100–200 mg quantities for isolation purposes and rely on chemical lysis or bead beating methods to isolate anaerobic fungal RNA/DNA are considered to be inadequate (Wang et al., 2011). With popular bead-beating based techniques the impact of the beads is cushioned by the fiber matrix, causing incomplete lysis of anaerobic fungal cells. If such methods are used, however, it is important to pre-process samples using mechanical grinding either in combination with freeze-drying or liquid N, as hand grinding is inadequate (Wang et al., 2011). Ground samples not only enable a better extraction of DNA from the microbes associated with the fiber matrix, but also allow a more representative ruminal sample to be extracted. This is important when only milligram quantities are used for extraction. Recently, Elekwachi et al. (2017) optimized a liquid N grinding/TRIzol extraction method which enabled effective lysis of anaerobic fungal cells, and resulted in a representative proportion of anaerobic fungi being detected in the sequence data subsequently generated. Application of this extraction method in future metagenomic and metatranscriptomics studies should, therefore, result in a more accurate understanding of the contribution of anaerobic fungi to the rumen microbiome.

Anaerobic fungi are often considered to play a limited role in the rumen when animals are fed diets supplemented with cereals. However, due to some anaerobic fungi having amylolytic activity, mixed effects of grain supplementation on anaerobic fungal numbers have been reported in the literature (Gordon and Phillips, 1998). Furthermore, fermentation of certain types of carbohydrates has also been shown to be strain specific (Trinci et al., 1994; Orpin and Joblin, 1997). Based on this information it is perhaps, therefore, not surprising that the type of anaerobic fungi enriched from rumen content samples was found to vary with the type of carbon source used (Griffith et al., 2009). Combined with the recent contrasting transcriptomic findings with respect to the influence of substrate type on the expression of CAZymes (section Gene expression analysis of axenic/monoxenic anaerobic fungal cultures), it is clear that there is an urgent need to rethink our understanding of anaerobic fungi as simply “fiber degraders.” There is now a clear evidence base supporting the concept of niche differentiation of anaerobic fungi in response to carbon source (Griffith et al., 2009), as well as the type of herbivorous host (Liggenstoffer et al., 2010).

As all the currently described Neocallimastigomycota belong to just one family, it is perhaps not entirely unexpected that there are increasing reports of anaerobic fungi in novel herbivorous hosts such as the iguana (Mackie et al., 2004; Liggenstoffer et al., 2010), sea urchin (Thorsen, 1999), and termite (Lee et al., 2015). More interestingly, however, there are increasing reports of anaerobic fungi being detected in non-gut environments using molecular and/or microscopy based techniques (Lockhart et al., 2006; Mohamed and Martiny, 2011; Ivarsson et al., 2016; Wurzbacher et al., 2016). Whilst some of these reports may be explainable by contamination of the environment by herbivorous hosts, it has recently been suggested that a novel lineage may exist within phylum Neocallimastigomycota (Picard, 2017). Until viable anaerobic fungal cultures are isolated from these non-gut environments, however, the ability of anaerobic fungi to exist in a free-living form will always be treated with skepticism. Perhaps to some extent this has a feeling of *déjà vu*, when 40 years ago the first reports of an anaerobic fungus challenged the dogma of the time.

To date the long term availability of anaerobic fungal cultures has been an issue, with several type strains being lost over the years. This is due to the inability of commercial culture collections to propagate anaerobic fungal cultures. Therefore, if a culture is deposited in a collection, it is only as good as the shelf-life of the corresponding cryovial. This is a problem that anaerobic fungal researchers are actively seeking to resolve through improving cryopreservation methods (Solomon et al., 2016b) and developing a centralized non-commercial repository of anaerobic fungal type strains and/or well characterized cultures within the anaerobic fungal research community.

Anaerobic fungal cultures will always be essential to our ability to understand the biology of anaerobic fungi, particularly as there is an increasing need to link ‘omics data with activity and physiology. This is even more important now that it is recognized that a large proportion of anaerobic fungal sequences cannot be functionally annotated, including highly expressed “core” genes. In order to help deliver improved models for future annotations, databases need to be (a) promptly updated with sequenced anaerobic fungal genomes as well as (b) strategies developed to characterize the function of the currently non-annotated “core” genes.

## Conclusions

Anaerobic fungi are a central component of the rumen microbiome, and are well established in terms of their key role in ruminal fiber degradation. Application of PCR based approaches in the past decade has increased our understanding of their diversity, and highlighted that many novel taxa remain to be cultivated. Understanding of their ecological role in the rumen is, however, undergoing a paradigm shift in light of the increasing evidence base indicating that anaerobic fungi have undergone substantial niche differentiation. There is a need to move beyond the broad generalization of anaerobic fungi as fiber-degraders, and explore the fundamental differences that underpin their ability to exist in distinct ecological niches. Application of genomics, transcriptomics, proteomics and metabolomics to their study in pure/mixed cultures and environmental samples will be invaluable in this process. A more central key challenge however, is advancing our understanding of the biology of axenic cultures using the wide range of ‘omics approaches that have been successfully developed to date. A central problem for all analyses is the limited functional annotation of anaerobic fungal sequence data. There is, therefore, an urgent need to expand information held within publicly available reference databases. Once this challenge is overcome, along with improved rumen sample collection and extraction, the application of these techniques will be key in furthering our understanding of the ecological role and impact of anaerobic fungi.

## Author contributions

JE initiated and co-ordinated the writing project. All authors contributed text to the manuscript. All co-authors contributed to the development of the manuscript as a whole by giving constructive feedback on the manuscript during its preparation. All authors gave approval of the manuscript for publication.

### Conflict of interest statement

The authors declare that the research was conducted in the absence of any commercial or financial relationships that could be construed as a potential conflict of interest.
