# Supplementary material for: PCR and Omics Based Techniques to Study the Diversity, Ecology and Biology of Anaerobic Fungi: Insights, Challenges and Opportunities
Source: Front Microbiol. 2017 Sep 25;8:1657. doi: 10.3389/fmicb.2017.01657 (PMC5622200; doi:10.3389/fmicb.2017.01657)
Supplement: Supplementary file 1 [file Table1.docx]

*Supplementary Material

**PCR and ‘omics based techniques to study the diversity, ecology and biology of anaerobic fungi: insights, challenges and opportunities**

**Joan E. Edwards^*^, Robert J. Forster, Tony M. Callaghan, Veronika Dollhofer, Sumit S. Dagar, Yanfen Cheng, Jongsoo Chang, Sandra Kittelmann, Katerina Fliegerova, Anil K. Puniya , John K. Henske, Sean P. Gilmore, Michelle A. O’Malley, Gareth W. Griffith and Hauke Smidt**

**^*^Correspondence:** Dr Joan E. Edwards: [joan.edwards@wur.nl](mailto:joan.edwards@wur.nl)

**Supplementary Table 1:** cDNA library based studies of anaerobic fungal hydrolases.

| **Genus** | **Enzymes** | **GH family** | **References** |
| --- | --- | --- | --- |
|  |  |  |  |
| *Neocallimastix* | endoglucanases | 5 | Fujino et al., 1998; Zhou et al., 1994; Xue et al., 1992;  Aylward et al., 1999 |
|  | exoglucanases | *6* | Denman et al., 1996 |
|  | xylanases | *10, 11* | Black et al., 1994; Gilbert et al., 1992; Gobius et al., 2002;  Xue et al., 1995; Huang et al., 2005, Durand et al., 1996 & 1999 |
|  |  |  |  |
| *Piromyces* | β-glucosidases | *1, 3* | Harhangi et al., 2002; Steenbakkers et al., 2003 |
|  | endoglucanases | *5,9, 45, 48* | Eberhardt et al., 2000; Tsai et al., 2003; Steenbakkers et al., 2002a, Raghothama et al., 2001; Steenbakkers et al., 2002b |
|  | exoglucanases | *6* | Harhangi et al., 2003c; Tsai et al., 2003 |
|  | xylanases | *11* | Fanutti et al., 1995 |
|  |  |  |  |
| *Orpinomyces* | β-glucosidases | *1* | Li et al., 2004a |
|  | endoglucanases | *5* | Li et al., 1997a; Qiu et al., 2000; Chen et al., 1998 |
|  | exoglucanases | *6* | Li et al., 1997b; Chen et al., 1998 & 2003; Steenbakkers et al., 2001,  Li et al., 2004b |
|  | xylanases | *11* | Li et al., 1997a; Li et al., 2007 |
|  |  |  |  |

**Supplementary Table 2:** Predicted secretory proteins from anaerobic fungi.

| **Accession Number** | **Name** | **Organism** | **pI** | **MW** | **Reference** |
| --- | --- | --- | --- | --- | --- |
| Q9Y871 | feruloyl esterase B | *Piromyces equi* | 3.97 | 53470 | Fillingham et al., 1999 |
| AAD45376 | cinnamoyl ester hydrolase EstA | *Piromyces equi* | 3.97 | 53470 | Fillingham et al., 1999 |
| CAB69284 | unnamed protein product | *Piromyces equi* | 3.97 | 53470 | Fillingham and Gilbert, 2000 |
| ANS13829 | swol | *Orpinomyces sp.* | 4.02 | 61146 | Morrison et al., 2016 |
| AEX92706 | putative cellulase | *Neocallimastix patriciarum* | 4.05 | 83321 | Wang et al., 2011 |
| AFI47447 | beta-glucosidase | *Neocallimastix patriciarum* | 4.07 | 82941 | Chen et al., 2012a |
| AAL01213 | mannanase | *Orpinomyces sp.* | 4.27 | 62462 | Steenbakkers et al., 2001 |
| P55296 | beta-mannanase A | *Orpinomyces sp.* | 4.40 | 66007 | Fanutti et al., 1995 |
| CAA62968 | mannan endo-1,4-beta-mannosidase | *Piromyces sp.* | 4.40 | 66007 | Fanutti et al., 1995 |
| ANS13824 | BGL3 | *Orpinomyces sp.* | 4.43 | 81767 | Morrison et al., 2016 |
| AEX92705 | putative cellulase | *Neocallimastix patriciarum* | 4.44 | 74172 | Wang et al., 2011 |
| CAB92326 | endoglucanase 5A | *Piromyces equi* | 4.46 | 192852 | Eberhardt et al., 2000 |
| AEX92714 | putative cellulase | *Neocallimastix patriciarum* | 4.47 | 51238 | Wang et al., 2011 |
| P55298 | beta-mannanase C | *Orpinomyces sp.* | 4.50 | 62049 | Millward-Sadler et al., 1996 |
| CAA66134 | endo-1,4 beta-mannanase | *Piromyces sp.* | 4.50 | 62049 | Millward-Sadler et al., 1996 |
| AEX92717 | putative cellulase | *Neocallimastix patriciarum* | 4.52 | 52960 | Wang et al., 2011 |
| AAM94167 | exoglucanase Cel6A | *Piromyces equi* | 4.52 | 51737 | Harhangi et al., 2003a |
| AEX92709 | putative cellulase | *Neocallimastix patriciarum* | 4.53 | 51724 | Wang et al., 2011 |
| AAK20910 | non-catalytic protein 1 | *Piromyces equi* | 4.53 | 50549 | Freelove et al., 2001 |
| ANS13826 | Cel48 | *Orpinomyces sp.* | 4.54 | 81392 | Morrison et al., 2016 |
| AEX92715 | putative cellulase | *Neocallimastix patriciarum* | 4.54 | 52410 | Wang et al., 2011 |
| AAP30745 | beta-glucosidase Cel1C | *Piromyces sp.* | 4.54 | 74417 | Harhangi et al., 2003b,c |
| AAN76735 | Cel48A precursor | *Piromyces equi* | 4.56 | 82879 | Steenbakkers et al., 2002b |
| AEX92710 | putative cellulase | *Neocallimastix patriciarum* | 4.57 | 51189 | Wang et al., 2011 |
| AEX92722 | putative cellulase | *Neocallimastix patriciarum* | 4.58 | 82335 | Wang et al., 2011 |
| ABY52798 | 1,4-beta-D-glucan-cellobiohydrolase | *Piromyces rhizinflatus* | 4.58 | 54719 | Duan et al., 2008a |
| P55297 | beta-mannanase B | *Orpinomyces sp.* | 4.58 | 62206 | Millward-Sadler et al., 1996 |
| CAA66061 | endo-1,4 beta-mannanase | *Piromyces sp.* | 4.58 | 62206 | Millward-Sadler et al., 1996 |
| ADO33720 | exoglucanase | *Piromyces sp.* | 4.64 | 51683 | Thulasi et al., 2010a |
| AAL92497 | exoglucanase Cel6A | *Piromyces sp.* | 4.65 | 52054 | Harhangi et al., 2003a |
| AAD45834 | beta-glucosidase | *Orpinomyces sp.* | 4.68 | 73600 | Li et al., 2004a |
| AAL01212 | cellobiohydrolase II-like cellulase CelI | *Orpinomyces sp.* | 4.68 | 52274 | Steenbakkers et al., 2001 |
| AAN76734 | cellulase Cel48A precursor | *Piromyces sp.* | 4.69 | 81756 | Steenbakkers et al., 2002b |
| AAL01211 | cellobiohydrolase II-like cellulase CelH | *Orpinomyces sp.* | 4.74 | 52146 | Steenbakkers et al., 2001 |
| ACA65427 | xylose isomerase | *Orpinomyces sp.* | 4.75 | 42572 | Madhavan et al., 2009 |
| CAB92325 | endoglucanase 45A | *Piromyces equi* | 4.78 | 42402 | Eberhardt et al., 2000 |
| AEO51792 | cellulase | *Orpinomyces sp.* | 4.79 | 51041 | Liu et al., 2011a |
| AAD04193 | cellulase | *Orpinomyces sp.* | 4.79 | 51136 | Li et al., 1997b |
| AEX92708 | putative cellulase | *Neocallimastix patriciarum* | 4.79 | 51838 | Wang et al., 2011 |
| AAC49731 | endoglucanase precursor | *Orpinomyces joyonii* | 4.81 | 96182 | Liu et al., 1997 |
| AAB69092 | acetylxylan esterase | *Neocallimastix patriciarum* | 4.83 | 39480 | Dalrymple et al., 1997 |
| ABY52794 | endo-1,3-1,4-beta-glucanase | *Neocallimastix patriciarum* | 4.84 | 25906 | Duan et al., 2008b |
| AAM81967 | Cel9A precursor | *Piromyces sp.* | 4.86 | 84037 | Steenbakkers et al., 2002a |
| CAB76571 | xylose isomerase | *Piromyces sp.* | 4.88 | 42534 | Harhangi et al., 2003b |
| CAC34952 | beta-glucosidase | *Piromyces sp.* | 4.99 | 74142 | Harhangi et al., 2002 |
| AAC14690 | acetyl xylan esterase A | *Orpinomyces sp.* | 4.99 | 32963 | Blum et al., 1999 |
| AFG19713 | D-xylose isomerase | *Piromyces sp.* | 5.00 | 42503 | Wang et al., 2014 |
| AFG25770 | D-xylose isomerase | *Piromyces sp.* | 5.00 | 42503 | Wang and Dun, 2013 |
| AFK92914 | beta-1,3-1,4 glucanase A51 | *Neocallimastix patriciarum* | 5.01 | 25810 | Hung et al., 2012 |
| ABY52796 | endo-1,3-1,4-beta-glucanase | *Piromyces communis* | 5.03 | 25189 | Duan et al., 2008b |
| AAR97890 | cellulosomal serpin precursor | *Piromyces sp.* | 5.03 | 59037 | Steenbakkers, et al., 2006 |
| AAD04192 | lichenase | *Orpinomyces sp.* | 5.06 | 25796 | Chen et al., 1997 |
| O14412 | endo-beta-1,3-1,4 glucanase; | *Orpinomyces sp.* | 5.06 | 25796 | Chen et al., 1997 |
| Q12647 | endo-1,4-beta-glucanase B | *Neocallimastix patriciarum* | 5.14 | 53064 | Zhou et al., 1994 |
| AAO41704 | beta-glucosidase precursor | *Piromyces sp.* | 5.25 | 91489 | Steenbakkers et al., 2003 |
| AAD51054 | exocellobiohydrolase Cbh120 | *Piromyces rhizinflatus* | 5.29 | 48492 | Tsai et al., 2003 |
| AAQ09257 | lichenase | *Anaeromyces sp.* | 5.29 | 25006 | Chen and Hseu, 2004 |
| AAF14365 | endo-1,4-beta-xylanase | *Neocallimastix patriciarum* | 5.30 | 48114 | Liu et al., 1999a |
| Q9UV68 | endo-1,4-beta-xylanase C | *Neocallimastix patriciarum* | 5.30 | 48114 | Liu et al., 1999a |
| AFK92913 | beta-1,3-1,4 glucanase M2 | *Neocallimastix patriciarum* | 5.40 | 25926 | Hung et al., 2012 |
| ABY52795 | endo-1,4-beta-xylanase | *Piromyces communis* | 5.49 | 37491 | Duan et al., 2008c |
| AAB92679 | cellulase C | *Orpinomyces sp.* | 5.68 | 47672 | Li et al., 1997b |
| AEO51791 | endo-1,4-beta-xylanase | *Orpinomyces sp.* | 5.79 | 37521 | Liu et al., 2011b |
| ACL68347 | endo-1,4-beta-xylanase | *Neocallimastix patriciarum* | 5.80 | 70590 | Pai et al., 2010 |
| B8YG19 | bifunctional acetylxylan esterase/xylanase | *Neocallimastix patriciarum* | 5.80 | 70590 | Pai et al., 2010 |
| AAC09066 | 1,4-beta-D-glucan-cellobiohydrolase | *Orpinomyces sp.* | 5.84 | 48594 | Chen et al., 2003a |
| AIK03053 | cellobiohydrolase C7 | *Orpinomyces sp.* | 5.86 | 47591 | Chen et al., 2014 |
| AFK92916 | beta-1,3-1,4 glucanase A16 | *Neocallimastix patriciarum* | 5.94 | 25942 | Hung et al., 2012 |
| AFK92915 | beta-1,3-1,4 glucanase A13 | *Neocallimastix patriciarum* | 5.94 | 25876 | Hung et al., 2012 |
| AAT99015 | xylanase | *Neocallimastix frontalis* | 6.08 | 64240 | Huang et al., 2005 |
| AAB23454 | xylanase A | *Neocallimastix patriciarum* | 6.08 | 64209 | Gilbert et al., 1992 |
| CAB58524 | xylanase | *Neocallimastix patriciarum* | 6.08 | 64209 | Gilbert and Hazlewood, 1999 |
| P29127 | bifunctional endo-1,4-beta-xylanase A | *Neocallimastix patriciarum* | 6.08 | 64199 | Gilbert et al., 1992 |
| CAA46498 | xylanase A | *Neocallimastix patriciarum* | 6.08 | 64199 | Gilbert et al., 2005 |
| CAA62969 | endo-1,4-beta-xylanase | *Piromyces sp.* | 6.08 | 65923 | Fanutti et al., 1995 |
| ABY52797 | cellobiohydrolase | *Piromyces rhizinflatus* | 6.16 | 49098 | Duan et al., 2008a |
| AEX92712 | putative cellulase | *Neocallimastix patriciarum* | 6.24 | 49223 | Wang et al., 2011 |
| AAD51055 | exocellobiohydrolase precursor Cbh6 | *Piromyces rhizinflatus* | 6.32 | 49358 | Tsai et al., 2003 |
| AAD04194 | xylanase | *Orpinomyces sp.* | 6.94 | 37560 | Li et al., 1997a |
| AAQ09258 | cellulase | *Anaeromyces sp.* | 6.97 | 48727 | Chen and Hseu, 2004 |
| AAB92678 | cellulase A | *Orpinomyces sp.* | 6.99 | 48830 | Li et al., 1997b |
| AAB69090 | acetylxylan esterase | *Neocallimastix patriciarum* | 7.36 | 40468 | Dalrymple et al., 1997 |
| CAA57717 | endoxylanase | *Neocallimastix frontalis* | 7.40 | 64067 | Durand et al., 1996 |
| AAQ09256 | cellobiohydrolase C precursor | *Neocallimastix sp.* | 7.80 | 43775 | Chen and Hseu, 2004 |
| AAC09067 | 1,4-beta-D-glucan-cellobiohydrolase | *Orpinomyces sp.* | 7.99 | 44579 | Chen et al., 2003b |
| ABY52793 | cellobiohydrolase | *Neocallimastix patriciarum* | 8.00 | 44063 | Duan et al., 2008c |
| AEX92711 | putative cellulase | *Neocallimastix patriciarum* | 8.00 | 43703 | Wang et al., 2011 |
| AAS06905 | pyruvate formate lyase activating enzyme | *Neocallimastix frontalis* | 8.05 | 24781 | Gelius-Dietrich and Henze, 2004 |
| Q01490 | cyclophilin B | *Orpinomyces sp.* | 8.16 | 19676 | Chen et al., 1995 |
| AAD04195 | cyclophilin B precursor | *Orpinomyces sp.* | 8.16 | 19676 | Chen and Ljungdahl, 1995 |
| ABC47329 | cyclophilin B precursor | *Orpinomyces sp.* | 8.16 | 19704 | Chen et al., 2005 |
| AAC09228 | cellobiohydrolase II | *Orpinomyces sp.* | 8.16 | 44578 | Chen et al., 2003c |
| AAC49315 | cellobiohydrolase precursor | *Neocallimastix patriciarum* | 8.18 | 43718 | Denman et al., 1996 |
| ACI26721 | cellobiohydrolase | *Neocallimastix patriciarum* | 8.23 | 43119 | Pai and Liu, 2008 |
| AAQ93324 | cellobiohydrolase | *Neocallimastix frontalis* | 8.33 | 41872 | Wang and Hseu, 2004 |
| ABW04217 | endo-1,4-beta-xylanase | *Neocallimastix patriciarum* | 8.61 | 33909 | Liu et al., 2008 |
| A8TGA1 | endo-1,4-beta-xylanase | *Neocallimastix patriciarum* | 8.61 | 33909 | Liu et al., 2008 |

**Supplementary Table 3:** Predicted non-secretory proteins from anaerobic fungi.

| **Accession Number** | **Name** | **Organism** | **pI** | **MW** | **Reference** |
| --- | --- | --- | --- | --- | --- |
| AEX92720 | putative cellulase | *Neocallimastix patriciarum* | 3.97 | 56184 | Wang et al., 2011 |
| AAD02028 | exocellobiohydrolase precursor | *Piromyces rhizinflatus* | 4.11 | 51510 | Liu et al., 1999b |
| AEX92713 | putative cellulase | *Neocallimastix patriciarum* | 4.14 | 56254 | Wang et al., 2011 |
| AAL01214 | endo-glucanase CelJ | *Orpinomyces sp.* | 4.19 | 24904 | Steenbakkers et al., 2001 |
| AEX92718 | putative cellulase | *Neocallimastix patriciarum* | 4.33 | 79057 | Wang et al., 2011 |
| AAD43818 | endoglucanase precursor | *Piromyces rhizinflatus* | 4.40 | 62623 | Tsai et al., 2003 |
| AEX92721 | putative cellulase | *Neocallimastix patriciarum* | 4.51 | 50884 | Wang et al., 2011 |
| 5CXU_A | ferulic acid esterase | *Anaeromyces mucronatus* | 4.56 | 31046 | Gruninger et al., 2016 |
| 5CXX_A | ferulic acid esterase | *Anaeromyces mucronatus* | 4.56 | 31046 | Gruninger et al., 2016 |
| 5CXX_B | ferulic acid esterase | *Anaeromyces mucronatus* | 4.56 | 31046 | Gruninger et al., 2016 |
| 5CXX_C | ferulic acid esterase | *Anaeromyces mucronatus* | 4.56 | 31046 | Gruninger et al., 2016 |
| ADZ47894 | Fae1A | *Anaeromyces mucronatus* | 4.56 | 31046 | Qi et al., 2011 |
| AEX92707 | putative cellulase | *Neocallimastix patriciarum* | 4.65 | 49435 | Wang et al., 2011 |
| AAR08200 | CelA | *Neocallimastix patriciarum* | 4.67 | 53944 | Wang and Hseu, 2004 |
| AEX92719 | putative cellulase | *Neocallimastix patriciarum* | 4.68 | 85330 | Wang et al., 2011 |
| 3AYR_A | endoglucanase | *Piromyces rhizinflatus* | 4.70 | 43318 | Tseng et al., 2011 |
| 3AYS_A | endoglucanase | *Piromyces rhizinflatus* | 4.74 | 43260 | Tseng et al., 2011 |
| ADO33719 | cellulase | *Neocallimastix sp.* | 4.74 | 54000 | Thulasi et al., 2010b |
| AAB69348 | cellulase | *Orpinomyces joyonii* | 4.78 | 43830 | Qiu et al., 2000 |
| CAA76356 | ketol-acid reductoisomerase | *Piromyces sp.* | 4.89 | 39449 | Akhmanova, 1998 |
| CAB76572 | D-xylulokinase | *Piromyces sp.* | 4.95 | 53151 | Harhangi et al., 2003b |
| Q12647 | endo-1,4-beta-glucanase B | *Neocallimastix patriciarum* | 5.14 | 53064 | Zhou et al., 1994 |
| CAA83238 | endoglucanase B | *Neocallimastix patriciarum* | 5.14 | 53064 | Zhou et al., 1994 |
| AFJ73477 | hexokinase 4 | *Neocallimastix frontalis* | 5.20 | 51632 | Park et al., 2012 |
| AFJ73476 | hexokinase 3 | *Neocallimastix frontalis* | 5.21 | 51670 | Park et al., 2012 |
| AFJ73490 | phosphoglycerate mutase | *Neocallimastix frontalis* | 5.26 | 56905 | Park et al., 2012 |
| P42894 | Enolase | *Neocallimastix frontalis* | 5.30 | 47011 | Durand et al., 1995 |
| CBN61506 | unnamed protein product | *Neocallimastix frontalis* | 5.30 | 47011 | Plesch et al., 2010 |
| AFJ73492 | enloase | *Neocallimastix frontalis* | 5.30 | 47011 | Park et al., 2012 |
| CAA56645 | enolase | *Neocallimastix frontalis* | 5.30 | 47011 | Durand et al., 1995 |
| AFJ73493 | enolase | *Neocallimastix frontalis* | 5.31 | 47010 | Park et al., 2012 |
| AFJ73491 | phosphoglycerate mutase | *Neocallimastix frontalis* | 5.34 | 56765 | Park et al., 2012 |
| 3WP4_A | xylanase | *Neocallimastix patriciarum* | 5.37 | 24739 | Cheng et al., 2014 |
| AFJ73523 | homocitrate synthase | *Neocallimastix frontalis* | 5.42 | 44147 | Park et al., 2012 |
| AFJ73482 | fructose-bisphosphate aldolase | *Neocallimastix frontalis* | 5.63 | 39664 | Park et al., 2012 |
| AFJ73475 | hexokinase 2 | *Neocallimastix frontalis* | 5.63 | 50442 | Park et al., 2012 |
| AAK61605 | heat shock protein 60 precursor | *Neocallimastix patriciarum* | 5.64 | 64293 | van der Giezen et al., 2003 |
| AFJ73524 | homocitrate synthase | *Neocallimastix frontalis* | 5.65 | 46258 | Park et al., 2012 |
| 3WP6_A | xylanase | *Neocallimastix patriciarum* | 5.70 | 24681 | Cheng et al., 2014 |
| 3WP5_A | xylanase | *Neocallimastix patriciarum* | 5.70 | 24610 | Cheng et al., 2014 |
| AFJ73483 | fructose-bisphosphate aldolase | *Neocallimastix frontalis* | 5.71 | 39630 | Park et al., 2012 |
| 2VG9_A | Xyn11a | *Neocallimastix patriciarum* | 5.73 | 24076 | Vardakou et al., 2008 |
| AFJ73525 | homocitrate synthase | *Neocallimastix frontalis* | 5.76 | 46500 | Park et al., 2012 |
| AFJ73474 | hexokinase 1 | *Neocallimastix frontalis* | 5.82 | 50540 | Park et al., 2012 |
| AAC05164 | 1,4-beta-D-glucan-4-glucanohydrolase | *Orpinomyces sp.* | 5.85 | 53629 | Chen et al,, 1998 |
| AFJ73526 | homocitrate synthase | *Neocallimastix frontalis* | 5.90 | 46508 | Park et al., 2012 |
| AFJ73489 | phosphoglycerate kinase | *Neocallimastix frontalis* | 5.91 | 44749 | Park et al., 2012 |
| AFJ73484 | triose phosphate isomerase | *Neocallimastix frontalis* | 5.96 | 27063 | Park et al., 2012 |
| AFJ73494 | pyruvate kinase | *Neocallimastix frontalis* | 6.04 | 67243 | Park et al., 2012 |
| AAQ22352 | aldehyde/alcohol dehydrogenase | *Piromyces sp.* | 6.07 | 97332 | Boxma et al., 2004 |
| CAA76361 | malate dehydrogenase | *Piromyces sp.* | 6.10 | 33025 | Akhmanova et al., 1998 |
| CAA76360 | aconitate hydratase | *Piromyces sp.* | 6.18 | 82057 | Akhmanova et al., 1998 |
| AEH95579 | cellulobiohydrolase | *Orpinomyces sp.* | 6.18 | 38283 | Liu et al., 2013 |
| AFJ73503 | acetaldehyde dehydrogenase | *Neocallimastix frontalis* | 6.18 | 97439 | Park et al., 2012 |
| AFJ73520 | aconitate hydratase | *Neocallimastix frontalis* | 6.23 | 81914 | Park et al., 2012 |
| AFJ73504 | acetaldehyde dehydrogenase | *Neocallimastix frontalis* | 6.25 | 97348 | Park et al., 2012 |
| AFJ73488 | phosphoglycerate kinase | *Neocallimastix frontalis* | 6.27 | 44754 | Park et al., 2012 |
| AAB69347 | cellulase | *Orpinomyces joyonii* | 6.33 | 53658 | Qiu et al., 2000 |
| 2C1F_A | Xylanase | *Neocallimastix Patriciarum* | 6.38 | 25978 | Vardakou et al., 2008 |
| AFJ73508 | phosphoenolpyruvate carboxykinase | *Neocallimastix frontalis* | 6.41 | 66911 | Park et al., 2012 |
| CAA57820 | endoxylanase | *Neocallimastix frontalis* | 6.41 | 29685 | Durand et al., 1996 |
| AAC49572 | malic enzyme precursor | *Neocallimastix frontalis* | 6.44 | 65538 | van der Giezen et al., 1997 |
| AFJ73527 | malic enzyme | *Neocallimastix frontalis* | 6.44 | 65538 | Park et al., 2012 |
| P78715 | Malic enzyme | *Neocallimastix frontalis* | 6.44 | 65538 | van der Giezen et al., 1997 |
| AFJ73529 | malic enzyme | *Neocallimastix frontalis* | 6.44 | 65535 | Park et al., 2012 |
| AFJ73530 | malic enzyme | *Neocallimastix frontalis* | 6.44 | 65505 | Park et al., 2012 |
| AFJ73510 | malate dehydrogenase | *Neocallimastix frontalis* | 6.48 | 33047 | Park et al., 2012 |
| AFJ73511 | malate dehydrogenase | *Neocallimastix frontalis* | 6.48 | 33038 | Park et al., 2012 |
| AFJ73486 | glyceraldehyde-3-phosphate dehydrogenase | *Neocallimastix frontalis* | 6.49 | 35925 | Park et al., 2012 |
| ABG47413 | ornithine carbamoyltransferase | *Neocallimastix frontalis* | 6.50 | 37946 | Gelius-Dietrich et al., 2006 |
| P22130 | phosphoenolpyruvate carboxykinase | *Neocallimastix frontalis* | 6.55 | 66896 | Reymond et al., 1992 |
| AAA33553 | phosphoenolpyruvate carboxykinase | *Neocallimastix frontalis* | 6.55 | 66896 | Reymond et al., 1992 |
| AFJ73528 | malic enzyme | *Neocallimastix frontalis* | 6.71 | 65678 | Park et al., 2012 |
| AFJ73531 | malic enzyme | *Neocallimastix frontalis* | 6.72 | 65605 | Park et al., 2012 |
| AAP70004 | heat shock protein 70 precursor | *Neocallimastix patriciarum* | 6.73 | 71285 | van der Giezen et al., 2003 |
| AFJ73518 | isocitrate dehydrogenase | *Neocallimastix frontalis* | 6.75 | 46148 | Park et al., 2012 |
| AFJ73507 | alcohol dehydrogenase | *Neocallimastix frontalis* | 7.12 | 37585 | Park et al., 2012 |
| AFJ73485 | triose phosphate isomerase | *Neocallimastix frontalis* | 7.20 | 26868 | Park et al., 2012 |
| AEH95580 | cellulobiohydrolase | *Orpinomyces sp.* | 7.25 | 43319 | Liu et al., 2013 |
| AFJ73502 | pyruvate formate lyase activating enzyme | *Neocallimastix frontalis* | 7.42 | 30098 | Park et al., 2012 |
| AFJ73534 | hydrogenase | *Neocallimastix frontalis* | 7.87 | 70518 | Park et al., 2012 |
| AAK60409 | hydrogenase | *Neocallimastix frontalis* | 7.87 | 70441 | Voncken et al., 2002a |
| AFJ73487 | glyceraldehyde-3-phosphate dehydrogenase | *Neocallimastix frontalis* | 7.88 | 36109 | Park et al., 2012 |
| ADN44280 | cellobiohydrolase C8 | *Orpinomyces sp.* | 7.94 | 45925 | Chen et al., 2012b |
| AFJ73509 | phosphoenolpyruvate carboxykinase | *Neocallimastix frontalis* | 8.07 | 67450 | Park et al., 2012 |
| CAB4145 | beta-succinyl CoA synthetase precursor | *Neocallimastix frontalis* | 8.14 | 47148 | Brondijk et al., 1996 |
| P53587 | succinate-CoA ligase | *Neocallimastix frontalis* | 8.14 | 47148 | Brondijk et al., 1996 |
| AFJ73533 | iron hydrogenase precursor | *Neocallimastix frontalis* | 8.15 | 70814 | Park et al., 2012 |
| AAP33147 | heat shock protein 60 | *Piromyces sp.* | 8.25 | 64722 | Voncken et al., 2002b |
| AFJ73517 | succinyl-CoA synthase beta subunit precursor | *Neocallimastix frontalis* | 8.45 | 36388 | Park et al., 2012 |
| AFJ73478 | glucose-6-phosphate isomerase 1 | *Neocallimastix frontalis* | 8.47 | 63350 | Park et al., 2012 |
| AFJ73479 | glucose-6-phosphate isomerase 2 | *Neocallimastix frontalis* | 8.59 | 64163 | Park et al., 2012 |
| AOT98541 | paxillin | *Orpinomyces sp.* | 8.61 | 37872 | Calkins and Youssef, 2016 |
| AAP83351 | succinyl-CoA synthetase, beta subunit | *Neocallimastix patriciarum* | 8.66 | 47136 | Dacks et al., 2006 |
| AFJ73501 | pyruvate formate lyase | *Neocallimastix frontalis* | 8.85 | 89697 | Park et al., 2012 |
| AFJ73513 | malate dehydrogenase | *Neocallimastix frontalis* | 8.92 | 33471 | Park et al., 2012 |
| AFJ73499 | pyruvate formate lyase | *Neocallimastix frontalis* | 8.93 | 89377 | Park et al., 2012 |
| AFJ73512 | malate dehydrogenase | *Neocallimastix frontalis* | 8.94 | 33519 | Park et al., 2012 |
| ABF66349 | truncated xylanase | *Neocallimastix frontalis* | 9.06 | 26716 | Wang et al., 2006a |
| AFJ73500 | pyruvate formate lyase | *Neocallimastix frontalis* | 9.06 | 89102 | Park et al., 2012 |
| ABB90952 | elongation factor 1-alpha | *Neocallimastix sp.* | 9.21 | 43250 | James and Vilgalys, 2007 |
| ABF66350 | xylanase | *Neocallimastix frontalis* | 9.28 | 33017 | Wang et al., 2006b |
| AFJ73516 | succinyl-CoA synthase alpha subunit | *Neocallimastix frontalis* | 9.57 | 34382 | Park et al., 2012 |
| AAP13544 | succinyl-CoA synthetase alpha subunit | *Neocallimastix frontalis* | 9.74 | 34403 | Gelius-Dietrich and Henze, 2003 |
| AFJ73515 | succinyl-CoA synthase alpha subunit | *Neocallimastix frontalis* | 9.74 | 34403 | Park et al., 2012 |
| AAP83350 | succinyl-CoA synthetase, alpha subunit | *Neocallimastix patriciarum* | 9.93 | 35524 | Dacks et al., 2006 |

**References:**

Akhmanova, A., Voncken, F.G., Harhangi, H., Hosea, K.M., Vogels, G.D. and Hackstein, J.H. (1998). Cytosolic enzymes with a mitochondrial ancestry from the anaerobic chytrid *Piromyces* sp. E2. *Mol. Microbiol.* 30, 1017-1027. NCBI (https://www.ncbi.nlm.nih.gov/protein/CAA76356, CAA76360, CAA76361).

Aylward, J.H., Gobius, A.S., Xue, G.-P., Simpson, G.D., Dalrymple B.P. (1999). The *Neocallimastix patriciarum* cellulase, CelD, contains three almost identical domains with high specific activities on Avicel. *Enzyme Microb. Technol.* 24, 609-614.

Black, G.W., Hazlewood, G.P., Xue, G.P., Orpin, C.G., Gilbert, H.J. (1994). Xylanase B from *Neocallimastix patriciarum* contains a noncatalytic 455-residue linker sequence comprised of 57 repeats of an octapeptide. *Biochem. J.* 299, 381-387.

Blum, D.L., Li, X.L., Chen, H. and Ljungdahl, L.G. (1999). Characterization of an acetyl xylan esterase from the anaerobic fungus *Orpinomyces* sp. strain PC-2. *Appl. Environ. Microbiol.* 65, 3990-3995. NCBI (https://www.ncbi.nlm.nih.gov/protein/AAC14690).

Boxma, B., Voncken, F., Jannink, S., Van Alen, T., Akhmanova, A., Van Weelden, S.W., Van Hellemond, J.J., Ricard, G., Huynen, M., Tielens, A.G. and Hackstein, J.H. (2004). The anaerobic chytridiomycete fungus *Piromyces* sp. E2 produces ethanol via pyruvate: formate lyase and an alcohol dehydrogenase E. *Mol. Microbiol.* 51, 1389-1399. NCBI (https://www.ncbi.nlm.nih.gov/protein/AAQ22352).

Brondijk, T.H., Durand, R., van der Giezen, M., Gottschal, J.C., Prins, R.A. and Fevre, M. (1996). scsB, a cDNA encoding the hydrogenosomal beta subunit of succinyl-CoA synthetase from the anaerobic fungus *Neocallimastix frontalis*. *Mol. Gen. Genet.* 253, 315-323. NCBI (https://www.ncbi.nlm.nih.gov/protein/CAB41451, P53587).

Calkins, S, and Youssef, N. H. (2016). Insights into the Utility of the Focal Adhesion Scaffolding Proteins in the Anaerobic Fungus *Orpinomyces* sp. C1A. *PLoS ONE* **11**: E0163553. doi: 10.1371/journal.pone.0163553. NCBI (https://www.ncbi.nlm.nih.gov/protein/AOT98541).

Chen, H., Li, X.L. and Ljungdahl, L.G. (1995). A cyclophilin from the polycentric anaerobic rumen fungus *Orpinomyces* sp. strain PC-2 is highly homologous to vertebrate. *PNAS* 92, 2587-2591. NCBI (https://www.ncbi.nlm.nih.gov/protein/Q01490, AAD04195)

Chen, H., Li, X.L. and Ljungdahl, L.G. (1997). Sequencing of a 1, 3-1, 4-beta-D-glucanase (lichenase) from the anaerobic fungus *Orpinomyces* strain PC-2: properties of the enzyme expressed in *Escherichia coli* and evidence that the gene has a bacterial origin. *J. Bacteriol.* 179, 6028-6034. NCBI (https://www.ncbi.nlm.nih.gov/protein/AAD04192, O14412).

Chen, H., Li, X.L., Blum, D.L. and Ljungdahl, L.G. (1998). Two genes of the anaerobic fungus *Orpinomyces* sp. strain PC-2 encoding cellulases with endoglucanase activities may have arisen by gene duplication. *FEMS Microbiol. Lett.* 159, 63-68. NCBI (https://www.ncbi.nlm.nih.gov/protein/AAC05164).

Chen, H.Z., Li, X.-L. and Ljungdahl, L.G. (2003a). Cellobiohydrolase cDNAs from the polycentric rumen anaerobic fungus *Orpinomyces* sp. strain PC-2. NCBI (https://www.ncbi.nlm.nih.gov/protein/AAC09066).

Chen, H., Li, X.L., Blum, D.L., Ximenes, E.A. and Ljungdahl, L.G. (2003b). CelF of *Orpinomyces* PC-2 has an intron and encodes a cellulose (CelF) containing a carbohydrate-binding module. *Appl. Biochem. Biotechnol.* 105, 775-785. NCBI (https://www.ncbi.nlm.nih.gov/protein/AAC09067)

Chen, H.Z., Li, X.-L. and Ljungdahl, L.G. (2003c). NCBI (https://www.ncbi.nlm.nih.gov/protein/AAC09228).

Chen, Y. and Hseu, R. (2004). NCBI (https://www.ncbi.nlm.nih.gov/protein/AAQ09256, AAQ09257, AAQ09258).

Chen, H., Li, X.-L., Xu, H., Ljungdahl, L.G. and Cerniglia, C.E. (2005). High level expression and characterization of anaerobic fungus *Orpinomyces* sp. strain PC-2 cyclophilin B gene. NCBI (https://www.ncbi.nlm.nih.gov/protein/ABC47329).

Chen, H.L., Chen, Y.C., Lu, M.Y., Chang, J.J., Wang, H.T., Wang, T.Y., Ruan, S.K., Wang, T.Y., Hung, K.Y., Cho, H.Y., Ke, H.M., Lin, W.T., Shih, M.C. and Li, W.H. (2012a). A highly efficient beta-glucosidase from a buffalo rumen fungus *Neocallimastix patriciarum* W5. *Biotechnol. Biofuels* **5**: 24. doi: 10.1186/1754-6834-5-24. NCBI (https://www.ncbi.nlm.nih.gov/protein/AFI47447).

Chen, Y.-C., Wang, H.-Y. and Liu, J.-C. (2012b). NCBI (https://www.ncbi.nlm.nih.gov/protein/ ADN44280).

Chen, Y.C., Chen, W.T., Liu, J.C., Tsai, L.C. and Cheng, H.L. (2014). A highly active beta-glucanase from a new strain of rumen fungus *Orpinomyces* sp.Y102 exhibits cellobiohydrolase and cellotriohydrolase activities. *Bioresour. Technol.* 170C, 513-521. NCBI (https//www.ncbi.nlm.nih.gov/protein/AFI47447, AIK03053).

Cheng, Y.S., Chen, C.C., Huang, C.H., Ko, T.P., Luo, W., Huang, J.W., Liu, J.R. and Guo, R.T. (2014). Structural analysis of a glycoside hydrolase family 11 xylanase from *Neocallimastix patriciarum*: insights into the molecular basis of a thermophilic enzyme. *J. Biol. Chem.* 289, 11020-11028. NCBI (https://www.ncbi.nlm.nih.gov/protein/3WP4_A, 3WP5_A, 3WP6_A).

Dacks, J.B., Dyal, P.L., Embley, T.M. and van der Giezen, M. (2006). Hydrogenosomal succinyl-CoA synthetase from the rumen-dwelling fungus *Neocallimastix patriciarum*; an energy-producing enzyme of mitochondrial origin. *Gene* 373, 75-82. NCBI (https://www.ncbi.nlm.nih.gov/protein/ AAP83350, AAP83351).

Dalrymple, B.P., Cybinski, D.H., Layton, I., McSweeney, C.S., Xue, G.P., Swadling, Y.J. and Lowry, J.B. (1997). Three *Neocallimastix patriciarum* esterases associated with the degradation of complex polysaccharides are members of a new family of hydrolases. *Microbiology* 143, 2605-2614. NCBI (https://www.ncbi.nlm.nih.gov/protein/ AAB69090, AAB69092).

Denman, S., Xue, G. P. and Patel, B. (1996). Characterization of a *Neocallimastix patriciarum* cellulase cDNA (celA) homologous to *Trichoderma reesei* cellobiohydrolase II. *Appl. Environ. Microbiol.* 62, 1889-1896. NCBI (https://www.ncbi.nlm.nih.gov/protein/AAC49315).

Duan, C.-H., Liu, J.-R. and Cheng, K.-J. (2008a). Cloning of a cellobiohydrolase gene from rumen fungus and its expression in *Escherichia coli*. NCBI (https://www.ncbi.nlm.nih.gov/protein/ABY52797, ABY52798)

Duan, C.-H., Liu, J.-R. and Cheng, K.-J. (2008b). Cloning of a lichenase gene from rumen fungus and its expression in *Escherichia coli*. NCBI (https://www.ncbi.nlm.nih.gov/protein/ABY52794, ABY52796)

Duan, C.-H., Liu, J.-R. and Cheng, K.-J. (2008c). Cloning of a xylanase gene from rumen fungus and its expression in *Escherichia coli*. NCBI (https://www.ncbi.nlm.nih.gov/protein/ABY52793, ABY52795).

Durand, R., Fischer, M., Rascle, C. and Fevre, M. (1995). *Neocallimastix frontalis* enolase gene, enol: first report of an intron in an anaerobic fungus. *Microbiology* 141, 1301-1308. NCBI (https://www.ncbi.nlm.nih.gov/protein/ CAA56645, P42894).

Durand, R., Rascle, C. and Fevre, M. (1996). Molecular characterization of xyn3, a member of the endoxylanase multigene family of the rumen anaerobic fungus *Neocallimastix frontalis. Curr. Genet.* 30, 531-540. NCBI (https://www.ncbi.nlm.nih.gov/protein/CAA57717, CAA57820).

Durand, R., Rascle, C., and Fevre M. (1999). Expression of a catalytic domain of a *Neocallimastix frontalis* endoxylanase gene (xyn3) in *Kluyveromyces lactis* and *Penicillium roqueforti.* *Appl. Microbiol. Biotechnol.* 52, 208-214.

Eberhardt, R.Y., Gilbert, H.J. and Hazlewood, G.P. (2000). Primary sequence and enzymic properties of two modular endoglucanases, Cel5A and Cel45A, from the anaerobic fungus *Piromyces equi*. *Microbiology* 146, 1999-2008. NCBI (https://www.ncbi.nlm.nih.gov/protein/CAB92326, CAB92325).

Fanutti, C., Ponyi, T., Black, G.W., Hazlewood, G.P. and Gilbert, H.J. (1995). The conserved noncatalytic 40-residue sequence in cellulases and hemicellulases from anaerobic fungi functions as a protein docking domain. *J. Biol. Chem.* 270, 29314-29322. NCBI (https://www.ncbi.nlm.nih.gov/protein/CAA62969, P55296).

Fillingham, I.J., Kroon, P.A., Williamson, G., Gilbert, H.J. and Hazlewood, G.P. (1999). A modular cinnamoyl ester hydrolase from the anaerobic fungus *Piromyces equi* acts synergistically with xylanase and is part of a multiprotein cellulose-binding cellulase-hemicellulase complex. *Biochem. J.* 343, 215-224. NCBI (https://www.ncbi.nlm.nih.gov/protein/Q9Y871, AAD45376).

Fillingham, I.J. and Gilbert, H.J. (2000). Phenolic acid esterase and use thereof. NCBI (https://www.ncbi.nlm.nih.gov/protein/CAB69284).

Freelove, A. C., Bolam, D. N., White, P., Hazlewood, G. P. and Gilbert, H. J. (2001). A novel carbohydrate-binding protein is a component of the plant cell wall-degrading complex of *Piromyces equi*. *J. Biol. Chem.* 276, 43010-43017. NCBI (https://www.ncbi.nlm.nih.gov/protein/AAK20910).

Fujino, Y., Ogata, K., Nagamine, T. and Ushida, K. (1998). Cloning, sequencing, and expression of an endoglucanase gene from rumen anaerobic fungus *Neocallimastix frontalis* MCH3. *Biosci. Biotechnol. Biochem.* 62, 1795-1798.

Gelius-Dietrich, G. and Henze, K. (2003). An alpha SCS cDNA from the chytrid fungus *Neocallimastix frontalis*. NCBI (https://www.ncbi.nlm.nih.gov/protein/ AAP13544).

Gelius-Dietrich, G. and Henze, K. (2004). Pyruvate formate lyase (PFL) and PFL activating enzyme in the chytrid fungus *Neocallimastix frontalis*: a free-radical enzyme system conserved across divergent eukaryotic lineages. *J. Eukaryot. Microbiol*. 51, 456-463. NCBI (https://www.ncbi.nlm.nih.gov/protein/AAS06905).

Gelius-Dietrich, G., ter Braak, M. and Henze, K. (2006). Mitochondrial steps of arginine biosynthesis are conserved in the hydrogenosomes of the chytridiomycete *Neocallimastix frontalis*. NCBI (https://www.ncbi.nlm.nih.gov/protein/ ABG47413).

Gilbert, H. J., Hazlewood, G. P., Laurie, J. I., Orpin, C. G. and Xue, G. P. (1992). Homologous catalytic domains in a rumen fungal xylanase: evidence for gene duplication and prokaryotic origin. *Mol. Microbiol.* 6, 2065-2072. NCBI (https://www.ncbi.nlm.nih.gov/protein/AAB23454, P29127, CAA46498).

Gilbert, H.J. and Hazlewood, G.P. (1999). Recombinant xylanases. NCBI (https://www.ncbi.nlm.nih.gov/protein/ CAB58524).

Gobius, K.S., Xue, G.P., Aylward, J.H., Dalrymple, B.P., Swadling, Y.J., McSweeney, C.S., and Krause, D.O. (2002). Transformation and expression of an anaerobic fungal xylanase in several strains of the rumen bacterium *Butyrivibrio fibrisolvens*. *J. Appl. Microbiol.* 93, 122-133.

Gruninger, R.J., Cote, C., McAllister, T.A. and Abbott, D.W. (2016). Contributions of a unique beta-clamp to substrate recognition illuminates the molecular basis of exolysis in ferulic acid esterases. *Biochem. J.* 473, 839-849. NCBI (https://www.ncbi.nlm.nih.gov/protein/5CXU_A, 5CXX_A, 5CXX_B, 5CXX_C).

Harhangi, H. R., Steenbakkers,P. J., Akhmanova, A., Jetten, M. S., van der Drift, C. and Op den Camp, H. J.(2002). A highly expressed family 1 beta-glucosidase with transglycosylation capacity from the anaerobic fungus *Piromyces* sp. E2. *Biochim. Biophys. Acta* 1574, 293-303. NCBI (https://www.ncbi.nlm.nih.gov/protein/CAC34952).

Harhangi, H.R., Freelove, A.C.J., Ubhayasekera, W., van Dinther, M., Steenbakkers, P.J.M., Akhmanova, A.S et al. (2003a). Ce6A, a major exoglucanase from the cellulosome of the anaerobic fungi *Piromyces* sp. E2 and *Piromyces equi.* NCBI (https//www.ncbi.nlm.nih.gov/protein/AAM94167, AAL92497).

Harhangi, H.R., Akhmanova, A.S., Emmens, R., van der Drift, C., de Laat, W.T., van Dijken, J.P., et al. (2003b). Xylose metabolism in the anaerobic fungus *Piromyces* sp. strain E2 follows the bacterial pathway. *Arch. Microbiol.* 180, 134-141. NCBI (https//www.ncbi.nlm.gov/protein/AAP30745, CAB76571, CAB76572).

Harhangi, H.R., Akhmanova, A.S., Steenbakkers, P. J., Jetten, M. S., van der Drift, C. And Op den Camp, H. J. (2003c). Genomic DNA analysis of genes encoding (hemi-) cellulolytic enzymes of the anaerobic fungus *Piromyces* sp. E2. *Gene* 314, 73-80. NCBI (https//www.ncbi.nlm.gov/protein/AAP30745).

Huang, Y.H., Huang, C.T. and Hseu, R.S. (2005). Effects of dockerin domains on *Neocallimastix frontalis* xylanases. *FEMS Microbiol. Lett.* 243, 455-460. NCBI (https://www.ncbi.nlm.nih.gov/protein/AAT99015).

Hung, Y.L., Chen, H.J., Liu, J.C. and Chen, Y.C. (2012). Catalytic Efficiency Diversification of Duplicate beta-1, 3-1, 4-Glucanases from *Neocallimastix patriciarum* J11. *Appl. Environ. Microbiol.* 78, 4294-4300. NCBI (https://www.ncbi.nlm.nih.gov/protein/ AFK92913, AFK92914, AFK92915, AFK92916).

James, T.Y. and Vilgalys, R. (2007). NCBI (https://www.ncbi.nlm.nih.gov/protein/ ABB90952).

Li, X.L., Chen, H. and Ljungdahl, L.G. (1997a). Monocentric and polycentric anaerobic fungi produce structurally related cellulases and xylanases. *Appl. Environ. Microbiol.* 63, 628-635. NCBI (https://www.ncbi.nlm.nih.gov/protein/AAD04193, AAD04194).

Li, X.L., Chen, H. and Ljungdahl, L.G. (1997b). Two cellulases, CelA and CelC, from the polycentric anaerobic fungus *Orpinomyces* strain PC-2 contain N-terminal docking domains for a cellulase-hemicellulase complex. *Appl. Environ. Microbiol.* 63, 4721-4728. NCBI (https://www.ncbi.nlm.nih.gov/protein/AAB92679, AAB92678).

Li, X.L., Ljungdahl, L.G., Ximenes, E.A., Chen, H., Felix, C.R., Cotta, M.A. and Dien, B.S. (2004a).Properties of a recombinant beta-glucosidase from polycentric anaerobic fungus *Orpinomyces* PC-2 and its application for cellulose hydrolysis. *Appl. Biochem. Biotechnol.* 113-116, 233-250. NCBI (https//www.ncbi.nlm.gov/protein/AAD45834).

Li, X.L., Ximenes, E.A., Chen, H., et al. (2004b). “Cloning and sequencing of two highly homologous cellulase genes CelH and CelI from the anaerobic fungus *Orpinomyces* strain PC-2” in *Biotechnology of Lignocellulose Degradation and Biomass Utilization*, eds. K. Ohmiya, K. Sakka, S. Karita, et al. (Tokyo, Japan: Uni Publisher Co., LTD.) 638-641

Li, X.L., Skory, C.D., Ximenes, E.A., Jordan, D.B., Dien, B.S., Hughes, S.R., and Cotta, M.A. (2007). Expression of an AT-rich xylanase gene from the anaerobic fungus *Orpinomyces* sp. strain PC-2 in and secretion of the heterologous enzyme by *Hypocrea jecorina.* *Appl. Microbiol. Biotechnol.* 74, 1264-1275.

Liu, J.H., Selinger, L.B., Hu, Y.J., Moloney, M.M., Cheng, K.J. and Beauchemin, K.A. (1997). An endoglucanase from the anaerobic fungus *Orpinomyces joyonii*: characterization of the gene and its product. *Can. J. Microbiol.* 43, 477-485. NCBI (https://www.ncbi.nlm.nih.gov/protein/AAC49731)

Liu, J.H., Selinger, B.L., Tsai, C.F. and Cheng, K.J. (1999a). Characterization of a *Neocallimastix patriciarum* xylanase gene and its product. *Can. J. Microbiol.* 45, 970-974. NCBI (https://www.ncbi.nlm.nih.gov/protein/AAF14365, Q9UV68)

Liu, J.-H., Prenevost, K., Qiu, X., Selinger, L.B., McAllister, T.M. and Cheng, K.-J. (1999b). NCBI (https://www.ncbi.nlm.nih.gov/protein/AAD02028).

Liu, J.R., Duan, C.H., Zhao, X., Tzen, J.T., Cheng, K.J. and Pai, C.K. (2008). Cloning of a rumen fungal xylanase gene and purification of the recombinant enzyme via artificial oil bodies. *Appl. Microbiol. Biotechnol.* 79, 225-233. NCBI (https://www.ncbi.nlm.nih.gov/protein/ABW04217, A8TGA1).

Liu, T. (2011a). Cloning, sequencing and expression of the cellulase gene celB from the anaerobic rumen fungus *Orpinomyces* sp. strain LT-3 isolated from Chinese Holstein cattle in *E. coli*. NCBI (https://www.ncbi.nlm.nih.gov/protein/AEO51792).

Liu, T., Zou, Y.-J., Wang, L. and Yang, S.-S. (2011b). Cloning, sequencing and expression of the xylanase gene xynA from the anaerobic rumen fungus *Orpinomyces* sp. strain LT-3 isolated from Chinese Holstein cattle in *E. coli*. NCBI (https://www.ncbi.nlm.nih.gov/protein/AEO51791).

Liu, J.-C., Wang, H.-Y. Chen, Y.-C. (2013). NCBI (https://www.ncbi.nlm.nih.gov/protein/ AEH95579, AEH95580).

Madhavan, A., Tamalampudi, S., Ushida, K., Kanai, D., Katahira, S., Srivastava, A., Fukuda, H., Bisaria, V.S. and Kondo, A. (2009). Xylose isomerase from polycentric fungus *Orpinomyces*: gene sequencing, cloning, and expression in *Saccharomyces cerevisiae* for bioconversion of xylose to ethanol*. Appl. Microbiol. Biotechnol.* 82, 1067-1078. NCBI (https://www.ncbi.nlm.nih.gov/protein/ACA65427)

Millward-Sadler, S. J., Hall, J., Black, G. W., Hazlewood, G. P. and Gilbert, H. J. (1996). Evidence that the *Piromyces* gene family encoding endo-1, 4-mannaases arose through gene duplication. *FEMS Microbiol. Lett.* 141, 183-188. NCBI (https//www.ncbi.nlm.nih.gov/protein/ P55297, P55298, CAA66134, CAA66061).

Morrison, J. M., Elshahed, M. S. and Youssef, N. H. (2016). Defined enzyme cocktail from the anaerobic fungus *Orpinomyces* sp. strain CIA effectively releases sugars from pretreated corn stover and switchgrass. *Sci. Rep.* **6**: 29217. doi: 10.1038/srep29217. NCBI (https://www.ncbi.nlm.nih.gov/protein/ANS13824, ANS13826, ANS13829).

Pai, C.-K. and Liu, J.-R. (2008). Cloning of a cellobiohydrolase gene from rumen fungus and its expression in *Escherichia coli*. NCBI (https://www.ncbi.nlm.nih.gov/protein/ACI26721)

Pai, C.K., Wu, Z.Y., Chen, M.J., Zeng, Y.F., Chen, J.W., Duan, C.H., Li, M.L. and Liu, J.R. (2010). Molecular cloning and characterization of a bifunctional xylanolytic enzyme from *Neocallimastix patriciarum*. *Appl. Microbiol. Biotechnol*. 85, 1451-1462. NCBI (https://www.ncbi.nlm.nih.gov/protein/ACL68347, B8YG19).

Park, M., Lee, S.K., Park, H., Song, J., Kwon, M. and Chang, J. (2012). Research on potent enzyme production from anaerobic rumen fungus, *Neocallimastix frontalis* based on EST DB. NCBI (https://www.ncbi.nlm.nih.gov/protein/ AFJ73474, AFJ73475, AFJ73476, AFJ73477, AFJ73478, AFJ73479, AFJ73482, AFJ73483, AFJ73484, AFJ73485, AFJ73486, AFJ73487, AFJ73488, AFJ73489, AFJ73490, AFJ73491, AFJ73492, AFJ73493, AFJ73494, AFJ73499, AFJ73500, AFJ73501, AFJ73502, AFJ73503, AFJ73504, AFJ73507, AFJ73508, AFJ73509, AFJ73510, AFJ73511, AFJ73512, AFJ73513, AFJ73515, AFJ73516, AFJ73517, AFJ73518, AFJ73520, AFJ73523, AFJ73524, AFJ73525, AFJ73526, AFJ73527, AFJ73528, AFJ73529, AFJ73530, AFJ73531, AFJ73533, AFJ73534)

Plesch, G., Puzio, P., Blau, A., Herold, M.M., Wendel, B., Kamlage, B., Schauwecker, F. and Looser, R. (2010). Process for the production of fine chemicals. NCBI (https://www.ncbi.nlm.nih.gov/protein/ CBN61506).

Qi, M., Wang, P., Selinger, L.B., Yanke, L.J., Forster, R.J. and McAllister, T.A. (2011). Isolation and characterization of a ferulic acid esterase (Fae1A) from the rumen fungus *Anaeromyces mucronatus*. *J. Appl. Microbiol.* 110, 1341-1350. NCBI (https://www.ncbi.nlm.nih.gov/protein/ADZ47894 )

Qiu, X., Selinger, B., Yanke, L. and Cheng, K. (2000). Isolation and analysis of two cellulase cDNAs from *Orpinomyces joyonii*. *Gene* 245, 119-126. NCBI (https://www.ncbi.nlm.nih.gov/protein/ AAB69347, AAB69348).

Raghothama, S., Eberhardt, R.Y., Simpson, P., Wigelsworth, D., White, P., Hazlewood, G.P., Nagy, T., Gilbert, H.J., and Williamson, M.P. (2001). Characterization of a cellulosome dockerin domain from the anaerobic fungus *Piromyces equi*. *Nature Struct. Biol.* 8, 775-778.

Reymond, P., Geourjon, C., Roux, B., Durand, R. and Fevre, M. (1992). Sequence of the phosphoenolpyruvate carboxykinase-encoding cDNA from the rumen anaerobic fungus *Neocallimastix frontalis*: comparison of the amino acid sequence with animals and yeast. *Gene* 110, 57-63. NCBI (https://www.ncbi.nlm.nih.gov/protein/P22130, AAA33553).

Steenbakkers, P. J., Li, X. L., Ximenes, E. A., Arts, J. G., Chen, H., Ljungdahl, L. G. and Op Den Camp, H. J. (2001). Noncatalytic docking domains of cellulosomes of anaerobic fungi. *J. Bacteriol.* 183, 5325-5333. NCBI (https//www.ncbi.nlm.nih.gov/protien/ AAL01211, AAL01212, AAL01213, AAL01214)

Steenbakkers, P.J., Ubhayasekera, W., Goossen, H.J., van Lierop, E.M., van der Drift, C., Vogels, G.D., Mowbray, S.L. and Op den Camp, H.J. (2002a). An intron-containing glycoside hydrolase family 9 cellulase gene encodes the dominant 90 kDa component of the cellulosome of the anaerobic fungus *Piromyces* sp. strain E2*. Biochem. J.* 365, 193-204. NCBI (https://www.ncbi.nlm.nih.gov/protein/AAM81967)

Steenbakkers, P.J., Freelove, A., Van Cranenbroek, B., Sweegers, B.M., Harhangi, H.R., Vogels, G.D., Hazlewood, G.P., Gilbert, H.J. and Op den Camp, H.J. (2002b). The major component of the cellulosomes of anaerobic fungi from the genus *Piromyces* is a family 48 glycoside hydrolase. *DNA Seq.* 13, 313-320. NCBI (https//www.ncbi.nlm.nih.gov/protein/AAN76735, AAN76734).

Steenbakkers, P.J., Harhangi, H.R., Bosscher, M.W., van der Hooft, M.M., Keltjens, J.T., van der Drift, C., Vogels, G.D. and op den Camp, H. J. (2003). beta-Glucosidase in cellulosome of the anaerobic fungus *Piromyces* sp. strain E2 is a family 3 glycoside hydrolase*. Biochem. J.* 370, 963-970. NCBI (https://www.ncbi.nlm.nih.gov/protein/AAO41704)

Steenbakkers, P.J., Irving, J.A., Harhangi, H.R., Swinkels, W.J., Akhmanova, A., Dijkerman, R., van der Drift, C., Whisstock, J.C. and Op den Camp, H.J. (2006). Serpins in cellulosomes. NCBI (https://www.ncbi.nlm.nih.gov/protein/ AAR97890).

Thulasi, A., Vivekanandan, M., Chandrasekharaiah, M. and Lyju Jose, V. (2010a). *Piromyces* sp. glycoside hydrolase family 6 exoglucanase. NCBI (https://www.ncbi.nlm.nih.gov/protein/ADO33720).

Thulasi, A., Vivekanandan, M., Chandrasekharaiah, M. and Lyju Jose, V. (2010b). Cellulase gene (CelA) from the fungi *Neocallimastix* isolated from camel. NCBI (https://www.ncbi.nlm.nih.gov/protein/ADO33719).

Tsai, C.F., Qiu, X. and Liu, J.H. (2003). A comparative analysis of two cDNA clones of the cellulase gene family from anaerobic fungus *Piromyces rhizinflata*. *Anaerobe* 9, 131-140. NCBI (https://www.ncbi.nlm.nih.gov/protein/ AAD43818, AAD51054, AAD51055)

Tseng, C.W., Ko, T.P., Guo, R.T., Huang, J.W., Wang, H.C., Huang, C.H., Cheng, Y.S., Wang, A.H. and Liu, J.R. (2011). Substrate binding of a GH5 endoglucanase from the ruminal fungus *Piromyces rhizinflata*. *Acta Crystallogr. Sect. F Struct. Biol. Cryst. Commun.* 67, 1189-1194. NCBI (https://www.ncbi.nlm.nih.gov/protein/3AYR_A, 3AYS_A).

Van der Giezen, M., Rechinger, K.B., Svendsen, I., Durand, R., Hirt, R.P., Fevre, M., Embley, T.M. and Prins, R.A. (1997). A mitochondrial-like targeting signal on the hydrogenosomal malic enzyme from the anaerobic fungus *Neocallimastix frontalis*: support for the hypothesis that hydrogenosomes are modified mitochondria. *Mol. Microbiol.* 23, 11-21. NCBI (https://www.ncbi.nlm.nih.gov/protein/AAC49572, P78715).

Van der Giezen, M., Birdsey, G.M., Horner, D.S., Lucocq, J., Dyal, P.L., Benchimol, M., Danpure, C.J. and Embley, T.M. (2003). Fungal hydrogenosomes contain mitochondrial heat-shock proteins. *Mol. Biol. Evol.* 20, 1051-1061. NCBI (https://www.ncbi.nlm.nih.gov/protein/AAK61605, AAP70004).

Vardakou, M., Dumon, C., Murray, J.W., Christakopoulos, P., Weiner, D.P., Juge, N., Lewis, R.J., Gilbert, H.J. and Flint, J.E. (2008). Understanding the structural basis for substrate and inhibitor recognition in eukaryotic GH11 xylanases. *J. Mol. Biol.* 375, 1293-1305. NCBI (https://www.ncbi.nlm.nih.gov/protein/ 2C1F_A, 2VG9_A).

Voncken, F.G., Boxma, B., van Hoek, A.H., Akhmanova, A.S., Vogels, G.D., Huynen, M., Veenhuis, M. and Hackstein, J.H. (2002a). A hydrogenosomal [Fe]-hydrogenase from the anaerobic chytrid *Neocallimastix* sp. L2. *Gene* 284, 103-112. NCBI (https://www.ncbi.nlm.nih.gov/protein/AAK60409).

Voncken, F., Boxma, B., Tjaden, J., Akhmanova, A., Huynen, M., Verbeek, F., Tielens, A.G., Haferkamp, I., Neuhaus, H.E., Vogels, G., Veenhuis, M. and Hackstein, J.H. (2002b). Multiple origins of hydrogenosomes: functional and phylogenetic evidence from the ADP/ATP carrier of the anaerobic chytrid *Neocallimastix* sp. *Mol. Microbiol.* 44, 1441-1454. NCBI (https://www.ncbi.nlm.nih.gov/protein/AAP33147).

Wang, H.C. and Hseu, R.S. (2004). NCBI (https://www.ncbi.nlm.nih.gov/protein/ AAR08200, AAQ93324).

Wang, Y., Yao, B. and Huang, H. (2006a). NCBI (https://www.ncbi.nlm.nih.gov/protein/ABF66349).

Wang, Y., Yao, B., Huang, H., and Fan, Y. (2006b). NCBI (https://www.ncbi.nlm.nih.gov/protein/ABF66350).

Wang, T.Y., Chen, H.L., Lu, M.Y., Chen, Y.C., Sung, H.M., Mao, C.T., et al. (2011). Functional characterization of cellulases identified from the cow rumen fungus *Neocallimastix patriciarum* W5 by transcriptomic and secretomic analyses. *Biotechnol Biofuels* **4**: 24. doi: 10.1186/1754-6834-4-24 NCBI (https://www.ncbi.nlm.nih.gov/protein/ AEX92705, AEX92706, AEX92707, AEX92708, AEX92709, AEX92710, AEX92711, AEX92712, AEX92713, AEX92714, AEX92715, AEX92717, AEX92718, AEX92719, AEX92720, AEX92721, AEX92722,).

Wang, Z. and Dun, B.-Q. (2013). NCBI (https://www.ncbi.nlm.nih.gov/protein/ AFG25770).

Wang, Z., Dun, B. and Lu, M. (2014). NCBI (https://www.ncbi.nlm.nih.gov/protein/ AFG19713).

Xue, G.P., Gobius, K.S., and Orpin, C.G. (1992). A novel polysaccharide hydrolase cDNA (celD) from *Neocallimastix patriciarum* encoding three multi-functional catalytic domains with high endoglucanase, cellobiohydrolase and xylanase activities. *J. Gen. Microbiol.* 138, 2397-2403.

Xue, G.P., Denman, S.E., Glassop, D., Johnson, J.S., Dierens, L.M., Gobius, K.S., and Aylward, J.H. (1995). Modification of a xylanase cDNA isolated from an anaerobic fungus *Neocallimastix patriciarum* for high level expression in *Escherichia coli*. *J. Biotechnol.* 38, 269-277.

Zhou, L., Xue, G.P., Orpin, C.G., Black, G.W., Gilbert, H.J. and Hazlewood, G.P. (1994). Intronless celB from the anaerobic fungus *Neocallimastix patriciarum* encodes a modular family A endoglucanase. *Biochem. J.* 297, 359-364. NCBI (https://www.ncbi.nlm.nih.gov/protein/ CAA83238, Q12647)
